# Long-Term Effects of a Web-Based Low-FODMAP Diet Versus Probiotic Treatment for Irritable Bowel Syndrome, Including Shotgun Analyses of Microbiota: Randomized, Double-Crossover Clinical Trial

**DOI:** 10.2196/30291

**Published:** 2021-12-14

**Authors:** Dorit Vedel Ankersen, Petra Weimers, Mette Bennedsen, Anne Birgitte Haaber, Eva Lund Fjordside, Moritz Emanuel Beber, Christian Lieven, Sanaz Saboori, Nicolai Vad, Terje Rannem, Dorte Marker, Kristine Paridaens, Suzanne Frahm, Lisbeth Jensen, Malte Rosager Hansen, Johan Burisch, Pia Munkholm

**Affiliations:** 1 Department of Gastroenterology North Zealand University Hospital Frederikssund Denmark; 2 Unseen Bio Anpartsselskab Copenhagen Denmark; 3 Ferring International Center Société Anonyme Saint-Prex Switzerland; 4 Department of Dietetics Herlev University Hospital Herlev Denmark

**Keywords:** irritable bowel syndrome, web-based low-FODMAP diet, probiotics, randomized trial, web-based, IBS, symptom management, treatment outcomes, outcomes, treatment, microbiota, microbiome, gastroenterology, mobile app, mHealth, eHealth

## Abstract

**Background:**

The long-term management of irritable bowel syndrome (IBS) poses many challenges. In short-term studies, eHealth interventions have been demonstrated to be safe and practical for at-home monitoring of the effects of probiotic treatments and a diet low in fermentable oligosaccharides, disaccharides, monosaccharides, and polyols (FODMAPs). IBS has been linked to alterations in the microbiota.

**Objective:**

The aim of this study was to determine whether a web-based low-FODMAP diet (LFD) intervention and probiotic treatment were equally good at reducing IBS symptoms, and whether the response to treatments could be explained by patients’ microbiota.

**Methods:**

Adult IBS patients were enrolled in an open-label, randomized crossover trial (for nonresponders) with 1 year of follow-up using the web application IBS Constant Care (IBS CC). Patients were recruited from the outpatient clinic at the Department of Gastroenterology, North Zealand University Hospital, Denmark. Patients received either VSL#3 for 4 weeks (2 × 450 billion colony-forming units per day) or were placed on an LFD for 4 weeks. Patients responding to the LFD were reintroduced to foods high in FODMAPs, and probiotic responders received treatments whenever they experienced a flare-up of symptoms. Treatment response and symptom flare-ups were defined as a reduction or increase, respectively, of at least 50 points on the IBS Severity Scoring System (IBS-SSS). Web-based ward rounds were performed daily by the study investigator. Fecal microbiota were analyzed by shotgun metagenomic sequencing (at least 10 million 2 × 100 bp paired-end sequencing reads per sample).

**Results:**

A total of 34 IBS patients without comorbidities and 6 healthy controls were enrolled in the study. Taken from participating subjects, 180 fecal samples were analyzed for their microbiota composition. Out of 21 IBS patients, 12 (57%) responded to the LFD and 8 (38%) completed the reintroduction of FODMAPs. Out of 21 patients, 13 (62%) responded to their first treatment of VSL#3 and 7 (33%) responded to multiple VSL#3 treatments. A median of 3 (IQR 2.25-3.75) probiotic treatments were needed for sustained symptom control. LFD responders were reintroduced to a median of 14.50 (IQR 7.25-21.75) high-FODMAP items. No significant difference in the median reduction of IBS-SSS for LFD versus probiotic responders was observed, where for LFD it was –126.50 (IQR –196.75 to –76.75) and for VSL#3 it was –130.00 (IQR –211.00 to –70.50; *P*>.99). Responses to either of the two treatments were not able to be predicted using patients’ microbiota.

**Conclusions:**

The web-based LFD intervention and probiotic treatment were equally efficacious in managing IBS symptoms. The response to treatments could not be explained by the composition of the microbiota. The IBS CC web application was shown to be practical, safe, and useful for clinical decision making in the long-term management of IBS. Although this study was underpowered, findings from this study warrant further research in a larger sample of patients with IBS to confirm these long-term outcomes.

**Trial Registration:**

ClinicalTrials.gov NCT03586622; https://clinicaltrials.gov/ct2/show/NCT03586622

## Introduction

Irritable bowel syndrome (IBS) is a functional gastrointestinal disorder that affects 10% to 20% of the population in westernized countries [1]. The main gastrointestinal symptoms include bloating, pain, diarrhea, constipation, and altered bowel habits; psychological comorbidity is common with IBS, thereby symptoms are often accompanied by coexisting conditions like stress, anxiety, and depression that, together, greatly impact patients’ quality of life (QoL) [1-3]. Studies have shown that approximately 60% [4] of IBS onset is associated with psychosocial stressors, and up to 32% [5] is associated with prior acute gastroenteritis. The pathophysiology of IBS is not fully understood, but some of the underlying mechanisms include altered gastrointestinal motility, visceral hypersensitivity, psychosocial disturbance, low-grade inflammation, altered gut-brain function, and microbial ecosystem dysbiosis [3,6].

Managing IBS continues to be challenging because of the complexity of its chronicity, heterogeneous patient groups with and without comorbidities, and a lack of both diagnostic tools and well-documented treatment strategies for long-term purposes. Treatment strategies for IBS range from lifestyle and dietary advice to pharmacological solutions that generally target only the primary symptom. Recently, holistic eHealth solutions that monitor symptoms and support patients with digestive diseases have proven valuable in involving and empowering patients, and are used as an adjuvant to current treatment regimens [7,8]. Ideally, an app for IBS would include dietary education supported by clinical dieticians, since dietary triggers are reported to be central to symptom generation in 50% to 84% of patients with IBS [7,9].

An exclusionary diet low in fermentable oligosaccharides, disaccharides, monosaccharides, and polyols (FODMAPs) has proven effective in reducing gastrointestinal symptoms, in particular abdominal pain, bloating, and diarrhea [10], with a response rate between 50% and 80% among patients with IBS [9]. In addition, a low-FODMAP diet (LFD) has been shown by some studies to improve the disease course of IBS [11,12] and inflammatory bowel disease (IBD) with IBS-like symptoms [11]. A previous study has shown that short-term, web-based management of IBS patients, while treating their symptoms with LFD and probiotics, is practical and effective [13]. Yet, the LFD has also been linked to a reduction in beneficial gastrointestinal bacteria [14] and to compromise nutritional intake, especially calcium [15]. Therefore, eventual reintroduction of foods high in FODMAPs is essential in long-term management [9].

The composition of gut microbiota differs between subgroups of IBS patients and healthy individuals [16]. This fact, together with an increasing awareness of the importance of a “healthy gut” and the gastrointestinal microbiome, has led to growing research, financial investment, and consumer interest in probiotic treatments that may provide benefits to patients with IBS [17,18]. Probiotic treatments are not currently included in recommended treatment strategies for IBS, but in some countries, national guidelines, such as those issued by NICE (the National Institute for Health and Care Excellence) in the United Kingdom [19], suggest that IBS patients could benefit from probiotic treatments for alleviating symptoms. However, the results for various probiotic solutions, as well as their dosing, duration, and efficacy as a treatment for IBS, are inconclusive [18,20,21]. Moreover, probiotic treatments do not seem to sustain any of the microbial changes they induce after cessation of treatment [22].

So far, no one has been able to fingerprint patients based on their fecal microbial composition and tailor a particular intervention for them individually [4].

The primary aim of this 1-year, web-based study was to determine whether a 4-week probiotic treatment, VSL#3 (Actial Farmaceutica Srl), and the LFD were equally as effective at reducing IBS symptoms, and whether the response to either of the two treatments could be explained by fecal microbiota. The study’s secondary aims were to evaluate patients’ QoL, the effect of multiple VSL#3 treatments in sustaining symptom control across 1 year, and reintroduction of FODMAPs among LFD responders, as well as to evaluate the web application IBS Constant Care (IBS CC) [23] for its efficacy in long-term clinical use.

## Methods

### Participants

Adult IBS patients, 18 years or older, fulfilling the Rome III criteria and subdiagnosed with either IBS-diarrhea (IBS-D) or IBS–mixed type (IBS-M) by a gastroenterologist were consecutively included in the study at North Zealand University Hospital (August 23, 2018, to October 18, 2019). Patients were excluded if they did not have a smartphone, had previously undergone gastrointestinal surgery, had been diagnosed with celiac disease or lactose intolerance, had been subdiagnosed with constipation or unspecified IBS, were pregnant, followed alternative diets, had a history of alcohol or drug abuse, had a BMI below 18.5 or above 35 (calculated as weight in kg divided by height in m^2^), had been diagnosed with any comorbidities (eg, diabetes), had previously been on an LFD guided by a dietician, had been on any probiotic or antibiotic treatment within the 3 months prior to inclusion, or had an IBS severity score lower than 175 at inclusion.

Healthy controls (HCs) older than 18 years, with a normal BMI (18.5-25), fecal calprotectin (FC) less than 70 mg/kg, and not taking daily medication or food supplements were recruited internally from our institution.

The Ethical Committee of Denmark (H-16023499) and the Danish Data Protection Agency (I-Suite No. 6262, NOH-2018-002) approved this study. The study was registered at ClinicalTrials.gov (NCT03586622; see Multimedia Appendix 1 for the CONSORT-EHEALTH checklist). All patients in the study gave written informed consent prior to their inclusion.

### Study Design and Protocol

#### Overview

**Figure 1:**
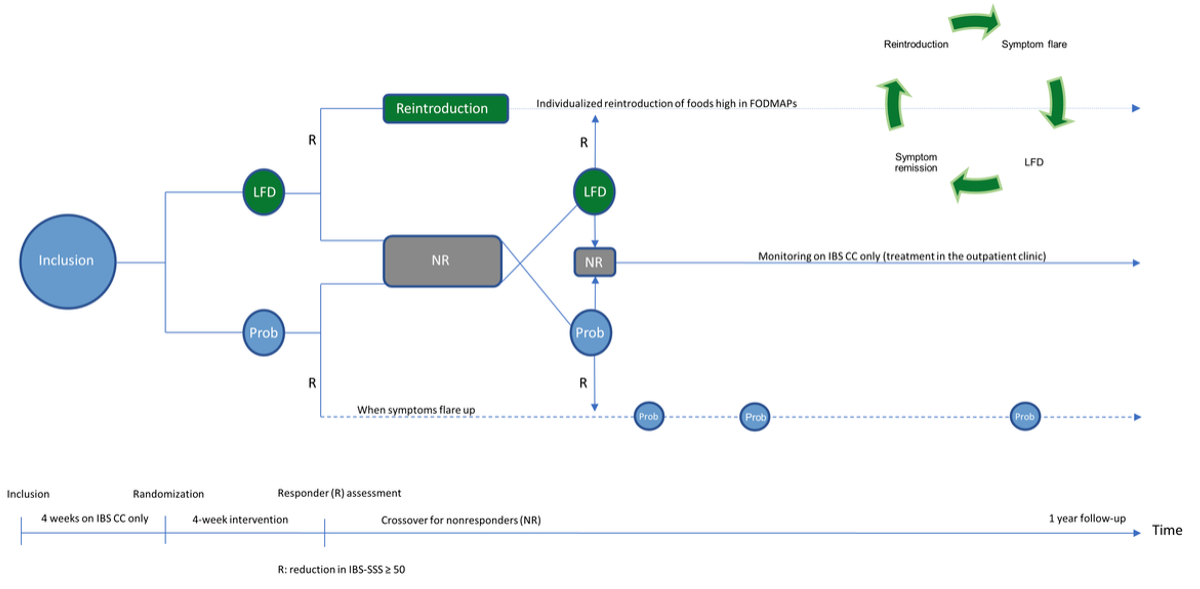
The 1-year design of this study. The IBS Constant Care (IBS CC) web application was used for home monitoring of symptoms and clinical decision making. Treatments included 4 weeks of monitoring on IBS CC, followed by randomization to either a 4-week low-FODMAP diet (LFD) or probiotic treatment (Prob; VSL#3, Actial Farmaceutica Srl). Responders (R) to the LFD would subsequently be reintroduced to foods higher in FODMAPs. Responders to probiotic treatment would receive multiple treatments upon symptom flare-ups (ie, an increase in the IBS Severity Scoring System [IBS-SSS] of more than 50 points). Response was defined as a decrease in IBS-SSS of at least 50 points. Nonresponders (NR) waited for a minimum of 2 weeks wash-out before being crossed over (this is not shown in the figure). FODMAPs: fermentable oligosaccharides, disaccharides, monosaccharides, and polyols; IBS: irritable bowel syndrome.

This eHealth study was an open-label, randomized, crossover trial (for nonresponders), with 1 year of follow-up (Figure 1). HCs home-monitored their weight, QoL, and symptoms at four points during the year using the IBS CC web application. These measurements were then represented in a traffic light format to the participants (see the Applications Used in the Study section for a description). At inclusion, patients were trained, for approximately 1 hour, in home monitoring of symptoms using IBS CC, including inflammation measured via FC. FC was measured using the CalproSmart app (Calpro AS). Patients were instructed to home-monitor every week for at least 4 weeks before randomization to either the LFD or probiotic treatment group.

Patients randomized to the LFD group had a consultation at the hospital with a nutritionist, who guided the patients individually through the diet’s principles and how to monitor their symptoms (see Multimedia Appendix 2 for home monitoring and screening intervals). Patients randomized to the probiotic treatment group were instructed on how to consume the sachets and home-monitor using IBS CC during treatment (Multimedia Appendix 2). After 4 weeks of either the LFD or probiotic treatment, patients’ response to treatment was evaluated. Response to treatment was defined as a reduction of at least 50 points in the IBS Severity Scoring System (IBS-SSS) [24]. If patients responded to the LFD, they were scheduled for a consultation on how to reintroduce high-FODMAP foods using IBS CC (described in Multimedia Appendix 2). Patients were instructed to resume a strict LFD for a few days when they encountered any symptom flare-ups (ie, an increase of at least 50 points in their IBS-SSS) during this reintroduction period (Figure 1). If patients responded to probiotic treatment, they were instructed to home-monitor their symptoms every week; if or when they relapsed (ie, saw an increase of at least 50 points in their IBS-SSS), they would receive another 4-week probiotic treatment, and so on, until 1 year of follow-up had elapsed (Figure 1). If patients did not respond to the initial treatment, they were instructed to take a wash-out period of at least 2 weeks, all the while continuing to home-monitor on IBS CC, and were subsequently crossed over. If patients did not respond to either of the two treatments, they could still home-monitor using IBS CC, following treatment advice from the outpatient clinic at the Department of Gastroenterology, North Zealand University Hospital. Web-based ward rounds were performed daily by the study investigator, based on an electronic patient list (green: inactive symptoms; yellow: mild to moderate symptoms; or red: severe symptoms, according to the IBS-SSS). Instructions to patients regarding how often they should monitor themselves at home using the IBS CC web application are listed in Multimedia Appendix 2, and email reminders (ie, “time to home-monitor”) were sent to participants that accepted these.

#### Fecal Sampling

Participants (IBS and HCs) were trained at inclusion in downloading the CalproSmart app and performing the FC home test. Participants received FC kits, including pads, frame, and tubes with buffer fluid, and were instructed to perform an FC home test within the first 4 weeks of home monitoring and again after 1 year of follow-up.

Participants were provided with easy sampler kits and were instructed to either send fecal samples for microbiota analysis via their general practitioner, or to store fecal samples in their domestic freezer (–20 ◦C). Participants (IBS and HCs) delivered their frozen fecal samples to the study investigator whenever they had an on-site consultation at the hospital. All samples were immediately frozen (–80 ◦C) upon receipt at the hospital. Patients were instructed to store or send fecal samples at study inclusion, at randomization, after 4 weeks of follow-up, and after 1 year of follow-up. Patients responding to probiotic treatment were further instructed to store samples every time they received a new 4-week probiotic treatment, sampled before and just after the treatment (Figure 1). HCs were instructed to collect a fecal sample four times during the course of 1 year and to store these samples in their domestic freezer. All fecal samples were shipped on dry ice to the microbiome laboratory in Germany.

### Interventions

#### Low-FODMAP Diet and Reintroduction of High-FODMAP Foods

One hour of dietary advice was provided on an individual basis by an experienced dietician or nutritionist that took into account the dietary history of each patient. Participants received guidance for the LFD using the Danish Low FODMAP Diet app (Muusmann Publisher), and were further instructed to measure their QoL, weight, adherence to the diet, and any symptoms on the IBS CC web application. In addition, participants also received supplementary materials (prepared by the study authors) on the LFD, with suggestions for LFD-friendly meals and recipes (eg, bread and ice cream); these documents were uploaded to each individual patient’s folder on IBS CC. Patients were advised to significantly reduce their intake of foods high in FODMAPs. Patients were advised only to consume foods low in FODMAPs, which were labeled green in the Low FODMAP Diet app; only two yellow-labeled foods, in small quantities, were allowed per day for reluctant participants.

Responders in the LFD group were taught to reintroduce foods high in FODMAPs using the food preference approach, all while taking into account their usual, preferred diet (described in Multimedia Appendix 2). 

#### Probiotic Treatment

VSL#3, distributed by Ferring Inc in Europe, is a high-concentration multi-strain probiotic mix containing one strain of *Streptococcus thermophilus* BT01, three strains of Bifidobacteria (*B breve* BB02; *B animalis* subspecies [subsp] *lactis* BL03, previously identified as *B longum* BL03; and *B* *animalis* subsp *lactis* BI04, previously identified as *B infantis* BI04), and four strains of Lactobacilli (*L acidophilus* BA05, *L plantarum* BP06, *L paracasei* BP07, and *L helveticus* BD08, previously identified as *L delbrueckii* subsp *bulgaricus* BD08). VSL#3 contains no less than 450 billion colony-forming units (CFU) per sachet. This formulation is sold as an over-the-counter food supplement at pharmacies in Denmark and by a variety of commercial websites.

VSL#3 sachets used in this study were provided by Actial Farmaceutica Srl, Italy, with the following batch numbers: 804033 and 908081. VSL#3 was administered for 26 days (approximately 4 weeks) for subjects allocated to the treatment group. Patients were instructed to take one sachet twice daily and store the sachets in the refrigerator. Patients were advised not to consume VSL#3 together with any hot or carbonated drinks. Side effects, if any, were registered throughout the study period.

### Measures

#### Applications Used in the Study

IBS CC has previously been used in other web-based studies [13,25]. The updated and expanded version of IBS CC [23] used in this study includes various patient-reported outcome measures (PROMs), listed in Multimedia Appendix 2 and described in detail below. Patient-reported satisfaction with the current version of IBS CC was evaluated before randomization and at 1-year follow-up. Participants gained access to IBS CC via a log-in procedure that includes two-factor authentication. IBS CC meets the requirements of the European Union’s General Data Protection Regulation.

Participants made their FC measurements at home using the CE (Conformité Européenne)–marked CalproSmart app. This home test can be performed in 18 minutes and is integrated into the IBS CC application, providing patients with an opportunity to see FC and other measures, such as the IBS-SSS, the Bristol Stool Chart (BSC), bowel movement frequency, weight and BMI, and QoL, all longitudinally and in a traffic light form. Two examples of a 1-year disease course, from a responder in the probiotic group and another in the LFD group, in IBS CC are shown in Multimedia Appendix 2.

The Low FODMAP Diet app is available from the App Store or Google Play and was provided free of charge to the patients in this study. The app includes sections about IBS, the LFD, recipes, grocery lists, a diary, and a module illustrating foods in green, yellow, and red. This mobile app is not integrated with IBS CC.

#### IBS Severity and Responder Definition

IBS severity was measured using the IBS-SSS [24]. This questionnaire consists of five items (ie, distention, bowel habits, interference with QoL, and two questions about abdominal pain) that are to be rated from 0 to 100 on a visual analog scale (VAS), with a total score ranging from 0 to 500. The IBS-SSS was made available on IBS CC with permission from Mapi Research Trust, Lyon, France. For ease of interpretation and patient involvement with IBS CC, a score of 0 to 175 was classified as “symptom remission and mild activity” (green), 175 to 300 as “moderate activity” (yellow), and 300 or more as “severe activity” (red). These cutoffs have been used in previous IBS web intervention trials [13,25]. Responses to an intervention or a symptom flare-up were defined as a change in IBS-SSS of at least 50 points [24].

#### Bristol Stool Chart and Bowel Movement Frequency

The BSC illustrates seven different stool types that represent constipation (types 1 and 2), “normal” stools (types 3-5), and diarrhea (types 6 and 7) [9]. Patients were instructed to assess their stool type either daily or weekly, together with bowel movement frequency, using the IBS CC web application. Results were illustrated via IBS CC to the patients longitudinally, with types 1 and 2 and types 6 and 7 in red, and “normal” stool types in green.

#### Adherence to Treatments

The FODMAP Adherence Report Scale (FARS) and the Medical Adherence Report Scale (MARS) measured adherence to the diet and probiotic treatment, respectively. The FARS and the MARS are self-assessment questionnaires consisting of five questions that have been used previously in IBD web intervention trials [26,27], one IBS study [11], and one IBD-IBS retrospective study [11]. The total scores range from 0 to 25, where 25 indicates maximum adherence. A score of 22 or higher was considered as adherent and was indicated as green to the patients in IBS CC.

#### Quality of Life

QoL was measured using the IBS-QoL questionnaire [28] (courtesy of Mapi Research Trust, Lyon, France) via IBS CC. The IBS-QoL questionnaire consists of 34 items, each with a 5-point response scale, resulting in a maximum score of 170. Scores were transformed to a scale of 0 to 100, where scores of 0 to 49 were indicated with a red color and a score of 50 or above was indicated in green, meaning a better QoL.

#### Disease Course Types

The Copenhagen disease course types describe four different IBS disease courses:

- A: Mild IBS with indolent course
- B: Mild IBS with aggressive course
- C: Chronic IBS with continuous course
- D: Chronic IBS with intermittent course.

These IBS types have previously been used in IBS studies by Maagaard et al [11] and Weynants et al [12]. Our patients were instructed to choose the disease course type that best described their course at inclusion and at 1-year follow-up. These measures were accessible to the participants on IBS CC.

#### Other Measures Included in IBS CC

Patients also registered their weight in kilograms on a weekly basis at home, and this measure was presented to patients longitudinally, together with their BMI, in IBS CC. Patients were instructed to use their bathroom scale, either in the morning or evening, with or without clothes, as long as they did so consistently throughout the study. The number of probiotic treatments needed for sustaining symptom control and the time between probiotic treatments (in days) were evaluated during the course of 1 year for multiple responders. Participants evaluated their satisfaction with IBS CC, including FC home testing, before their randomization on a VAS from 1 to 10, where 10 was the greatest possible satisfaction. Patient satisfaction with the study and IBS CC at 1 year, including FC home testing, was assessed using an e-questionnaire in IBS CC that was prepared by the authors, consisting of nine “yes or no” questions and two other questions: one regarding the time used for home monitoring and the other asking for suggestions on improving IBS CC for future clinical use. Clinical metadata were exported from the IBS CC database for statistical analyses.

#### Microbiome Analysis

Fecal samples were analyzed by CeGaT GmbH in Tübingen, Germany. The method used by CeGaT GmbH is based on culture-independent, whole-genome, shotgun metagenomic, next-generation sequencing. Genomic DNA was extracted from the participants’ fecal samples using CeGaT GmbH’s proprietary protocol, including enzymatic cleavage of genomic DNA. Approximately 100 ng of DNA was used per sample for library preparation, which was performed with the Nextera DNA Flex Library Prep Kit (Illumina). Sequencing was performed on the NovaSeq 6000 Sequencing System (Illumina), which resulted in at least 10 million 2 × 100 bp paired-end sequencing reads (2 Gb) per sample. Demultiplexing of the sequencing reads was performed with bcl2fastq Conversion Software (version 2.20; Illumina) [29]. Adapters were trimmed with Skewer [30]. Taxonomic classification and sequence abundance were estimated by Unseen Bio anpartsselskab, Greater Copenhagen, using an in-house bioinformatics pipeline [31] consisting of quality control with FastQC [32] and fastp [33], removal of human DNA by mapping reads with Kraken 2 [34] to its human library, and a summary using MultiQC [35]. The remaining sequencing reads were then mapped to a human gut microbiome-specific reference database, MGnify [36], with Kraken 2 in order to estimate bacterial and archaeal sequence abundance. Sequence abundance counts were then re-estimated to the species level using Bracken [37].

### Statistical Analyses

Counts, means, medians, and IQRs were computed from the PROMs. Wilcoxon rank-sum tests, Wilcoxon signed-rank tests with continuity correction, or Dunn all-pair tests were performed to compare medians. Analysis of sequence counts or reads and visualization of results were performed in R (version 4.0.4; The R Foundation). A list of R packages used in this study can be found in Multimedia Appendix 2. Sequence counts were rarefied to the smallest common value of 15 M using uniform sampling without replacement from the phyloseq package [38]. Sequence abundance was used as a proxy for taxonomic abundance. The α and β diversity were assessed using the inverse Simpson index [39] and Bray-Curtis dissimilarity [40], respectively. A *P* value lower than .05 was considered significant. We used a uniform manifold approximation and projection (UMAP) [41] technique in order to visually inspect potential clustering of the Bray-Curtis dissimilarity reduced to two dimensions. We estimated differential sequence abundance using a β-binomial model provided by the R package, corncob [42], with false discovery rate (<0.1)–adjusted *P* values. Clinical outcomes were assessed in a post hoc analysis by linear mixed-effect models [43]. Patients diagnosed with other diseases during the course of the study were excluded from the analyses if the disease affected their results; otherwise, they were included.

## Results

### Participants and Study Course

**Figure 2:**
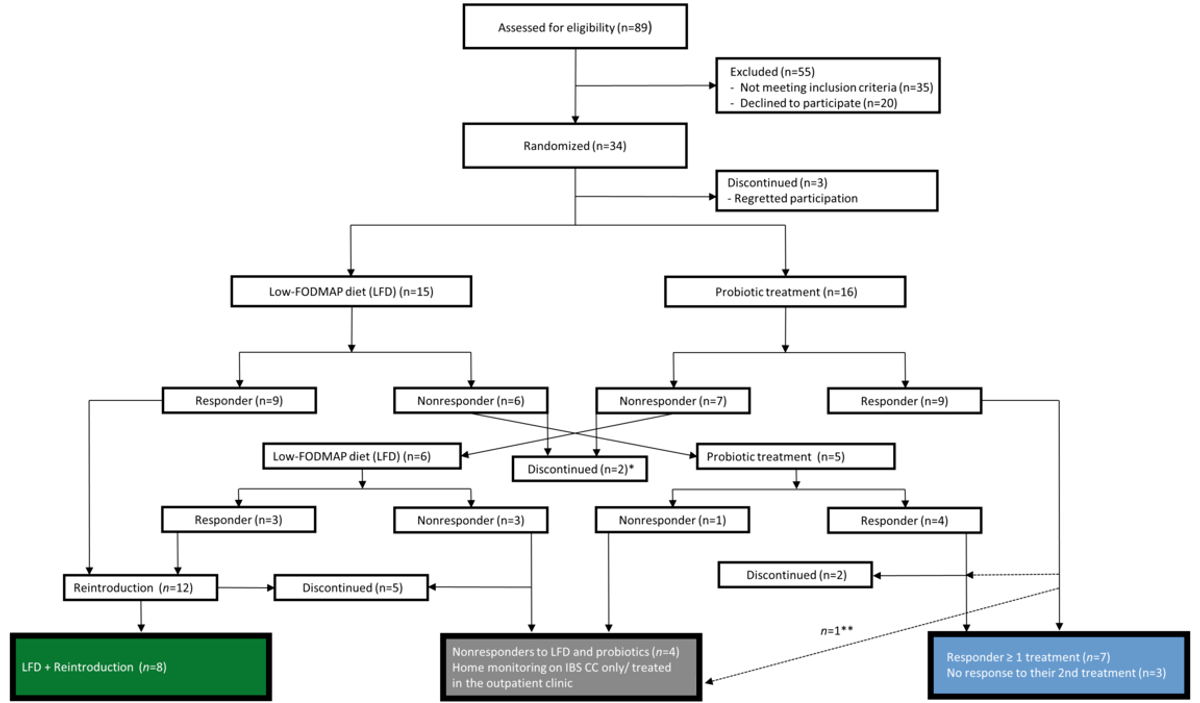
Flowchart of inclusion, individualized treatment response, and 1-year follow-up. Response to a 4-week probiotic treatment or LFD was defined as a decrease in the IBS Severity Scoring System (IBS-SSS) of at least 50 points. *One patient did not manage to cross over to probiotic treatment; however, this patient completed 1 year on the web application. This participant is labelled as "discontinued" in the figure. **Diagnosed with another disease and treated medically in the outpatient clinic. FODMAPs: fermentable oligosaccharides, disaccharides, monosaccharides, and polyols; IBS CC: IBS Constant Care.

In total, 34 IBS patients were included in the study (Figure 2). A total of 3 (9%) patients dropped out before randomization, and an additional 9 (26%) IBS patients discontinued participation during the course of the year. Reasons for discontinuation were divorce or other stress-related events. In total, 5 (15%) IBS patients were diagnosed with other diseases during the study: 1 with microscopic colitis, 1 with neuroendocrine tumor, 2 with bile acid malabsorption, and 1 with breast cancer; 4 of these completed the study. A total of 6 HCs were included, all of whom completed the 1-year study. Baseline data of participants are shown in Table 1. The number of IBS patients enrolled, randomizations to interventions, and responders and nonresponders to interventions can be found in Figure 2. Out of 21 patients, 12 responded to LFD (57%) and 8 (38%) completed the reintroduction to high-FODMAP foods. Out of 21 patients, 13 (62%) responded to their first VSL#3 treatment and 7 (33%) responded to multiple VSL#3 treatments. A total of 180 fecal samples (156 from IBS patients and 24 from HCs) were analyzed. Out of 180 fecal samples, 7 (3.9%) were removed from the metagenomic data set due to medical treatments undergone by the participants (ie, antibiotics or cholestyramine treatment). An additional 22 samples (12.2%) were removed from the data set for analyses that excluded patients diagnosed with other diseases during the study.

**Table 1:** Characteristics of participants at inclusion and upon randomization.

Characteristic			Inclusion (day 0; N=34 patients)	At randomization (week 4; n=31 patients)
**Irritable bowel syndrome (IBS) patients**				
	**IBS subtype, n (%)**			
		Mixed	15 (44)	15 (48)
		Diarrhea	19 (56)	16 (52)
	**Gender, n (%)**			
		Female	23 (68)	22 (71)
		Male	11 (32)	9 (29)
	**Smoking status, n (%)**			
		Never	18 (52)	18 (58)
		Current	4 (12)	4 (13)
		Previous	9 (26)	9 (29)
		Missing	3 (9)	0 (0)
	**Disease course type, n (%)**			
		A (mild IBS with indolent course)	0 (0)	0 (0)
		B (mild IBS with aggressive course)	11 (32)	11 (36)
		C (chronic IBS with continuous course)	16 (47)	16 (52)
		D (chronic IBS with intermittent course)	4 (12)	4 (13)
		Missing	3 (9)	0 (0)
	Age (years), median (IQR)		44.5 (27.5-51.5)	44 (26-50)
	Patient-reported years with IBS, median (IQR)		5 (2.9-15.0)	5 (2.5-15.0)
	BMI^a^, median (IQR)		23.9 (21.9-26.4)	23.6 (21.2-26.1)
	Fecal calprotectin (FC; mg/kg), median (IQR)		N/A^b^	53 (0-69)
	IBS-SSS^c^ score (0-500), median (IQR)		312 (243-370)	292 (225-356)
	Bristol Stool Chart score (1-7), median (IQR)		5 (3-6)	5 (3-6)
	Bowel movement frequency per day, median (IQR)		2 (1-5)	2 (2-4)
	IBS-QoL^d^ questionnaire score (0-100) , median (IQR)		54 (38-77)	53 (38-80)
	Evaluation of IBS CC^e^, including FC home test (1-10)^f^, median (IQR)		N/A	8 (7-9)
**Healthy controls (n=6)^g^**				
	**Gender, n (%)**			
		Female	5 (83)	N/A
		Male	1 (17)	N/A
	Age (years), median (IQR)		46 (30.8-58.5)	N/A
	BMI, median (IQR)		23.2 (20.8-25.1)	N/A
	IBS-SSS score (0-500), median (IQR)		13.5 (0.75-22.25)	N/A
	Bristol Stool Chart score (1-7), median (IQR)		4 (3.75-4.5)	N/A
	Bowel movement frequency per day, median (IQR)		1.5 (1-2.25)	N/A
	FC (mg/kg), median (IQR)		0 (0-72.75)	N/A
	IBS-QoL questionnaire score (0-100), median (IQR)		100 (98.25-100)	N/A

^a^BMI is calculated as weight in kg divided by height in m^2^.

^b^N/A: not applicable; data were not collected for this characteristic at this time point.

^c^IBS-SSS: IBS Severity Scoring System.

^d^IBS-QoL: IBS quality of life.

^e^IBS CC: IBS Constant Care.

^f^Satisfaction with the expanded IBS CC web application, including the FC home test, was evaluated by patients on a visual analog scale from 1 to 10, where 10 indicates the greatest possible satisfaction.

^g^Data for healthy controls were collected only at inclusion.

### Treatment Duration and Effect Sizes of the LFD and Probiotic Treatments Based on Response Types

The median time spent dieting by LFD responders (12/21, 57%) was 36 days (IQR 28.00-41.25), and the median time spent for reintroduction of high-FODMAP foods (8/21, 38%) was 296 days (IQR 227.00-305.75). For “true” responders (ie, participants who responded to multiple probiotic treatments [7/21, 33%]; see Figure 2), the median number of probiotic treatments needed for maintaining symptom control during the course of 1 year was 3 (IQR 2.25-3.75), and the median number of days between probiotic treatments was 46.5 (IQR 26.25-65.75). Symptom remission (ie, an IBS-SSS lower than 175) was achieved by 8 out of 12 (67%) LFD responders and 5 out of 7 (71%) multiple probiotic responders, the latter corresponding to 12 out of 19 (63%) VSL#3 treatments bringing about symptom remission in probiotic responders.

**Figure 3:**
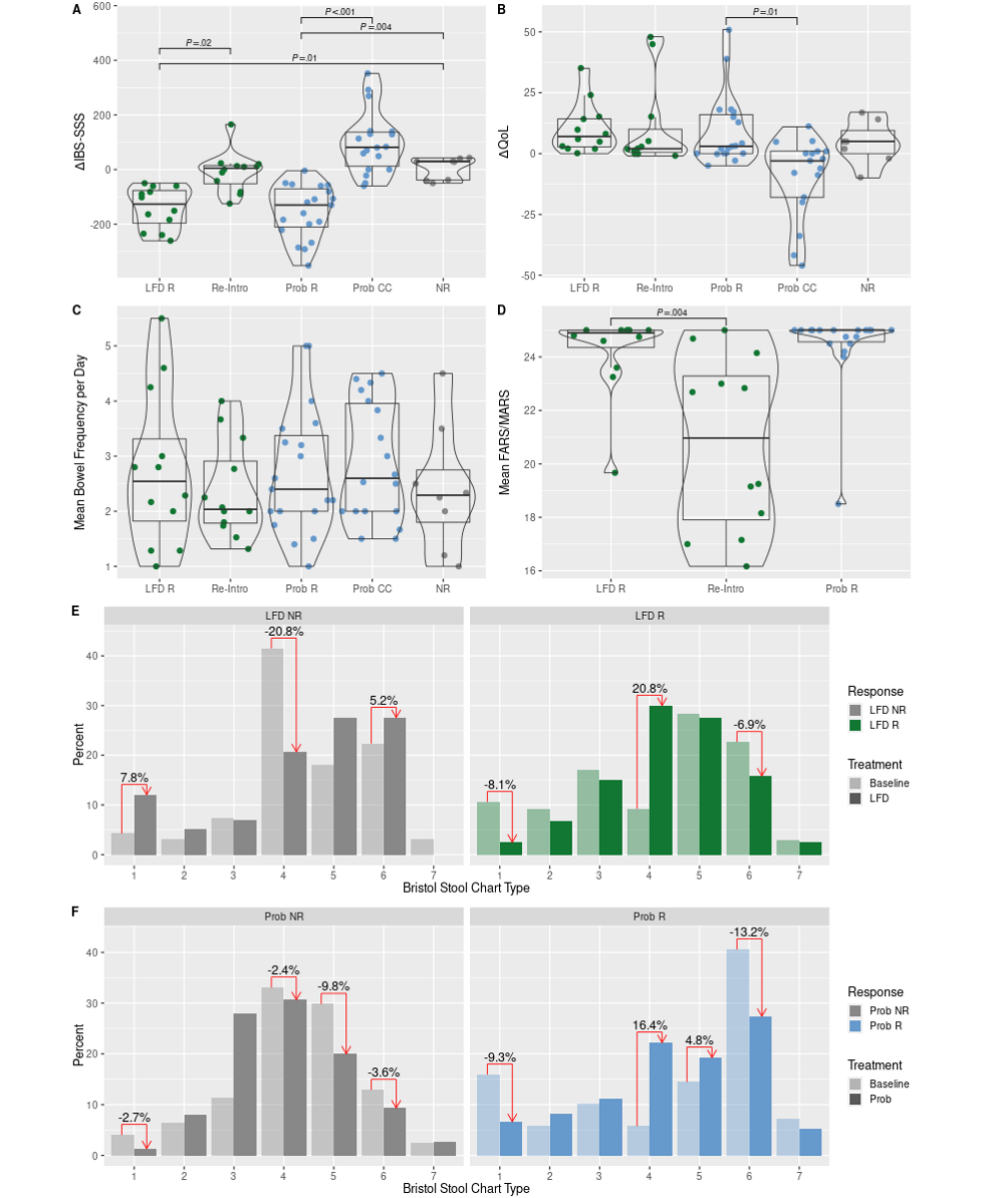
Effect sizes for select patient-reported outcome measures based on responder types. LFD responders (LFD R, n=12); reintroduction (Re-Intro, n=12); probiotic responders (Prob R, n=7; "true" responders; 19 treatments); in between active probiotic treatments (Prob CC, n=7; 18 between periods); nonresponders (NR, n=4). A. Change in IBS-SSS. B. Change in IBS-QoL. C. Mean bowel movement frequency per day. D. Mean adherence for LFD responders (FARS) and during reintroduction (FARS) and for probiotic responders (MARS). E and F. Change in Bristol Stool Chart scores, expressed as percentages. FARS: FODMAP Adherence Report Scale; FODMAPs: fermentable oligosaccharides, disaccharides, monosaccharides, and polyols; IBS: irritable bowel syndrome; IBS-SSS: IBS Severity Scoring System; LFD: low-FODMAP diet; MARS: Medical Adherence Report Scale; Prob: probiotic treatment; Prob CC: in between probiotic treatments, where patients are only measuring on IBS Constant Care (IBS CC; ie, not receiving any active treatments); QoL: quality of life; Re-Intro: resuming consumption of foods high in FODMAPs.

As shown in Figure 3, A, significant decreases in IBS-SSS were observed for LFD and probiotic responders relative to nonresponders; both responder types were adherent to treatments (Figure 3, D). However, no significant difference in effect size between the two treatments was found; the median IBS-SSS effect sizes were –126.50 (IQR –196.75 to –76.75) for LFD responders and –130.00 (IQR –211.00 to –70.50) for probiotic responders (*P*>.99). The corresponding changes in QoL among LFD and probiotic responders was not significant compared to nonresponders, nor were the median increases in QoL significant between the two responder groups, which were 7 (IQR 2.75-14.20) for LFD responders and 3 (IQR 0-16.00) for probiotic responders (*P*>.99). During reintroduction to high-FODMAP foods, patients deviated significantly from the principles of the LFD (Figure 3, D; *P*=.004), resulting in a small but significant median increase in symptom severity relative to the time period where they were on the LFD (Figure 3, A). In median terms, probiotic responders experienced a significant increase in symptom severity between active probiotic treatments, which also resulted in a significant median decrease in QoL (Figure 3, A and B). No significant changes in bowel movements per day based on responder type were observed (Figure 3, C). LFD and probiotic responders showed a tendency of normalizing stool appearance to type 4 (Figure 3, E and F). These results are generally substantiated by fitting linear mixed-effect models of clinical metadata from the IBS CC database (ie, not considering delta values alone) to estimate effect sizes (eg, for responder groups). These post hoc models of QoL showed that LFD responders saw significant increases in their QoL while on the LFD (by an estimated 9.08), and even more during reintroduction (13.48) relative to their baseline QoL (an estimated 60.20). The model estimating the effect on severity scoring showed significant decreases for all responder types, including probiotic responders between active treatments. Outputs from the linear mixed-effect models are shown in Multimedia Appendix 2.

No significant changes in FC based on responder type were registered between baseline and 1-year follow-up (Multimedia Appendix 2). At baseline, none of the IBS patients reported an indolent disease course type (ie, type A). At 1-year follow-up, 42% (5/12) and 43% (3/7) of the LFD and probiotic responders, respectively, reported an indolent disease course type (ie, type A). None of the nonresponders reported an indolent course at 1-year follow-up.

### Reintroduction of High-FODMAP Foods

A total of 8 out of 12 (67%) participants completed the process of reintroducing high-FODMAP foods (Figure 2) and succeeded in developing an individualized diet at 1-year follow-up. They reintroduced a median of 14.50 (IQR 7.25-21.75) high-FODMAP foods, of which they categorized a median of 7 (IQR 3-13) foods as green (ie, not symptom-triggering foods), while they categorized 5 (IQR 4-8) foods as yellow and 2 (IQR 1-4) foods as red (ie, symptom-triggering foods). Common among symptom-triggering foods were wheat and rye bread, pasta, pointed cabbage, onion, garlic, leek, broccoli, green peas, cauliflower, kidney beans, chickpeas, sweet potatoes, avocado, mushrooms, apples, and apple juice.

### Safety and Patient Satisfaction With IBS CC

No adverse events were registered for either intervention. The evaluation at 1-year follow-up showed overall satisfaction with the study, the support received during the study, and with IBS CC itself (further details can be found in Multimedia Appendix 2). During the 1-year study period, originating from both the personnel doing the daily web-based ward rounds and participants (n=33, including HCs), 887 web consultations and 781 text messages were registered.

### Healthy Controls

HCs did not receive any intervention during the study period and were in IBS-SSS remission at inclusion and at 1-year follow-up. No significant change was detected (median 13.5 [IQR 19.25] vs 15.5 [IQR 14.75], *P*=.79). They had high IBS-QoL scores at inclusion and 1-year follow-up (median 100 [0.75] vs 100 [0.75], *P*>.99). The primary purpose for the inclusion of HCs was to generate microbiome profiles that could serve as a reference.

### Basic Microbiota Descriptions in Relation to Diagnoses and Responder Types

A total of 10 million reads per sample were matched with high confidence to a median of 940 (IQR 18, range 821-1007) microbial species per sample. Median α diversity (measured by inverse Simpson index) was significantly greater for HCs (n=6) than for IBS patients (n=31), with median values of 51.60 (IQR 23.5) and 42.10 (IQR 16.1), respectively (*P*=.03; Multimedia Appendix 2). Across the entire study population, the Bray-Curtis dissimilarity within individuals was a median of 0.42 (IQR 0.14), and between individuals it was 0.72 (IQR 0.12; *P*<.001). There was also a significantly higher median of intraindividual Bray-Curtis dissimilarity in IBS-D compared to IBS-M patients (*P*=.03). UMAP plots based on IBS subtypes and responder types are shown in Multimedia Appendix 2. Those plots do not suggest any clusters, except for those given by the individuals themselves. The importance of individuals is also substantiated in the linear mixed-effect models estimating the change in α diversity for responder types (Multimedia Appendix 2). This model showed that approximately 24% of the variance was explained by the random effect of the individual and only 4% was explained by the fixed effect of responder type. In the model, nonresponders to the LFD had a lower inverse Simpson index that was close to significance (*P*=.07). No significant changes in α diversity for probiotic responders and nonresponders were found. Nonresponders to both probiotics and LFD showed a significantly higher median of intraindividual Bray-Curtis dissimilarity relative to probiotic responders (*P*=.01; Multimedia Appendix 2).

### Differential Analyses of Species Abundances Based on Responder Types

**Figure 4:**
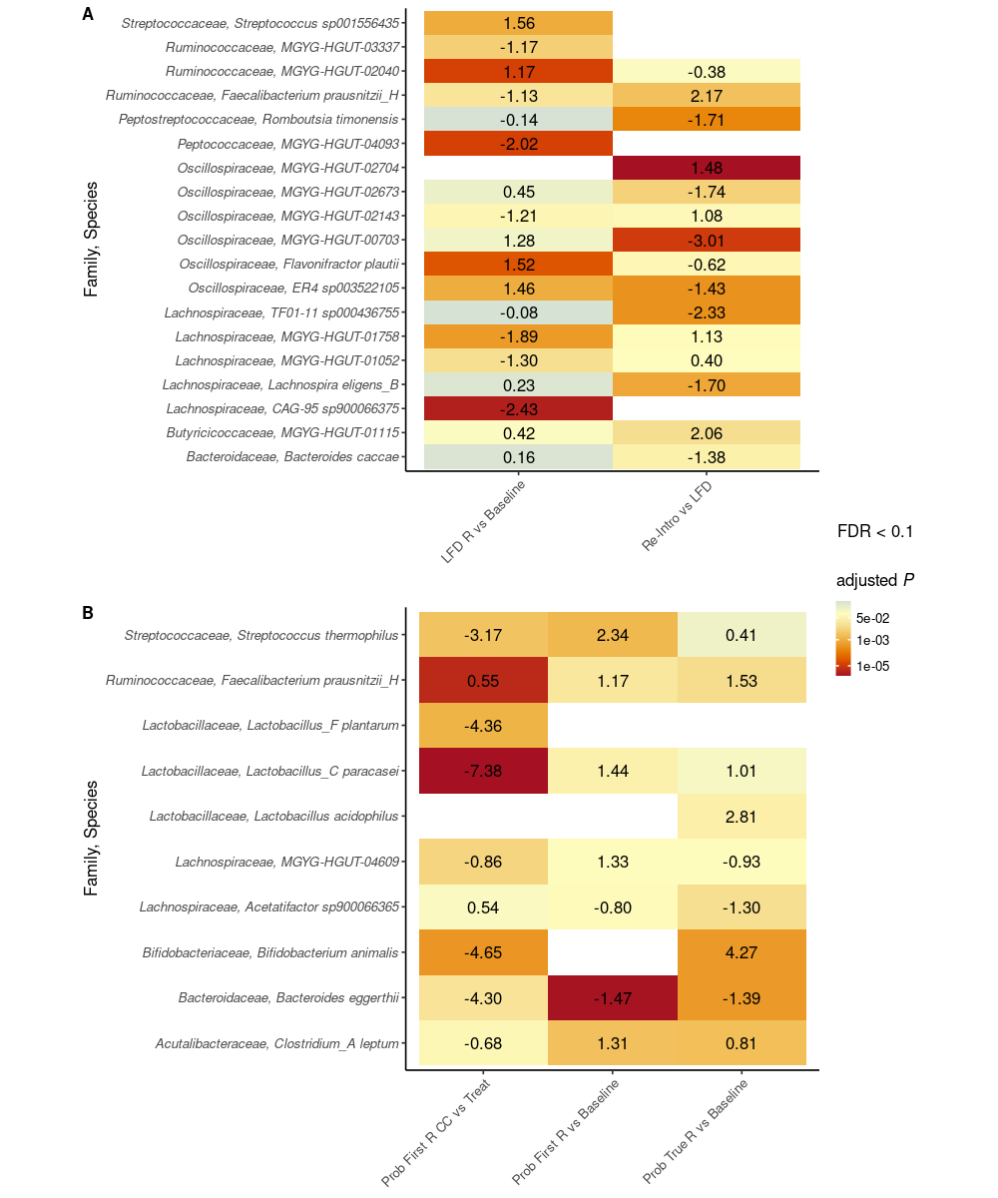
Heat maps showing the 10 most differentially abundant species for low-FODMAP diet (LFD) comparisons (A) and the top five species for the probiotic (Prob) comparisons (B). A. LFD responders (LFD R, n=12) and reintroduction (Re-Intro, n=8). B. Probiotic responders. First-time responders (Prob First R) to treatment (Treat) and the time between active treatments (the period in between active probiotic treatment is abbreviated as CC; n=13). Second-time treatment responders ("true" responders, Prob True R; n=7; 19 treatments). The values shown in the heat maps denote the expected difference in the logit-transformed relative abundances between two samples from the groups being compared, controlling for the effect of individuals. The color scale represents the false discovery rate (FDR) (<0.1)–adjusted *P* values. The scale ranges from red to yellow to blue. Yellow marks the *P* value .05. All *P* values smaller than .05 tend toward red, and those larger than .05 tend toward blue. FODMAPs: fermentable oligosaccharides, disaccharides, monosaccharides, and polyols.

No significant differential species abundances were found when comparing nonresponders to LFD and probiotic responders. Significant changes in relative species abundances induced by interventions (ie, LFD, reintroduction, and probiotic treatment) relative to baseline are shown in Figure 4 and further elaborated on in Multimedia Appendix 3. The corresponding Kyoto Encyclopedia of Genes and Genomes modules can be found in Multimedia Appendix 4.

## Discussion

### Principal Findings

In this study, we have shown that the new and expanded IBS CC web application for long-term management of IBS is practical, safe, and useful in clinical decision making. This was demonstrated by two year-long treatment interventions: the LFD (with subsequent reintroduction of high-FODMAP foods for LFD responders) and a probiotic treatment (repeated for 4 weeks every time a symptom flare-up occurred in probiotic responders). These therapeutics were found to be equally good at managing IBS symptoms in the short term and the long term. We also investigated participants’ fecal microbiota, in order to try to predict response to the interventions, in the hope of being able to individualize future treatments for IBS; however, we were unable to make these predictions. Due to the small sample size of the study, larger ones are needed to confirm our results and would, ideally, include other medical options for managing IBS, especially for nonresponders to the LFD and probiotic treatments.

### Sample Size and the Effect of the Individuals

Although the prevalence of IBS is high in westernized countries [1] such as Denmark, we were only able to include 34 out of an estimated target of 104 patients. This was mainly due to a high prevalence of comorbidities among eligible subjects that resulted in our excluding 64% of them. The high prevalence of comorbidities among eligible subjects is the topic of a newly published Danish nationwide study reporting that two-thirds of the Danish population, aged 16 years or older, suffer from one or more chronic conditions [44]. Our aim in excluding participants with multiple conditions, including other medical treatments, was to collect untarnished data for microbial analyses. The 5 patients with comorbidities or other diagnoses did not appear to affect the clinical data from IBS CC, nor did they affect the UMAP, inverse Simpson, or Bray-Curtis measures. However, these 5 patients did affect the results for the differential species abundance analyses based on responder types, possibly due to (1) the comorbidities themselves or (2) the fact that the individuals accounted for approximately 24% of the variance in the metagenomic data set. We aimed for including 104 IBS patients and 20 HCs. However, we did not manage to include our target population due to a high rate of co-morbidities among eligible subjects and implementation of new regulation (GDPR) in Denmark (data approval of the study took longer than expected).

### Probiotic Treatment

The response rates, adherence, effect sizes of median reductions in symptoms, and increases in QoL for probiotic and LFD responders were similar (ie, not significantly different); however, probiotic responders experienced a significant increase in median symptom severity and a decrease in QoL in the times between the 4-week probiotic treatments, as compared to the effect obtained during active probiotic treatment. However, the linear mixed-effect model estimated that probiotic “true” responders sustained significant symptom control in between their 4-week treatments relative to their first measure (ie, the intercept). This is also partly explained by the study design, which instructed probiotic responders to contact the study investigator for a new 4-week probiotic treatment upon experiencing a symptom flare-up (ie, an increase in their IBS-SSS of at least 50 points). Sustaining IBS symptom control in between active probiotic treatments has, to our knowledge, not been investigated before among “true” responders. The concept of sustaining symptom control among “true” responders—with a median of three 4-week treatments across the year-long study—and the timing of new probiotic treatments using eHealth, require further investigation in larger, clinical web-based trials. The high dose (2 × 450 billion CFU per day) and short duration combination used in this study seems reasonable, based on a meta-analysis that concluded that (1) probiotics are effective and safe for IBS patients (moderate evidence) and (2) single probiotics at a higher daily dose and for a shorter duration (less than 8 weeks) seem to be more efficacious (needs further evidence) [45]. Although the higher dose and shorter duration were related to single-strain probiotics, we have also documented the effect of multi-strain VSL#3. Among the top five most differentially abundant bacterial species found when comparing samples from probiotic responders to their baseline samples, we identified four species (ie, *S thermophilus*, *L paracasei*, *L acidophilus*, and *B animalis*) that are representative of strains contained in the VSL#3 mixture. Furthermore, all four species were completely absent from baseline samples, which means that the consumption of VSL#3 is the most plausible explanation for their presence. Future long-term studies need to investigate whether it is safe for patients to stop probiotic treatment after 4 weeks and to only resume when symptom flare-ups occur. Abrupt suspension of probiotics have been hypothesized to increase host pathogen susceptibility by inducing gut dysbiosis [46].

### LFD and Reintroduction of High-FODMAP Foods

Several key studies, meta-analyses, and reviews [10,47-50] have investigated the effects of an LFD on symptom reduction and changes in microbiota during an LFD [14,51-53]. Similar effect sizes and response rates to those we found have also been reported before [13,25,52]; however, the changes observed in the microbiota profile induced by the LFD in this study are not the same as reported in previous studies [14,51-53]. In one of these studies, easing of FODMAP restrictions (ie, reintroduction of foods high in FODMAPs) was recommended based on microbiota results indicating a significant decrease in total bacteria abundances, including significant changes in relative abundances of *Clostridium* cluster XIVa (reduced), *A muciniphila* (reduced), and *R torques* (increased) relative to a normal Australian diet [14]. Others have shown that the LFD, relative to sham diet advice, induces significant changes in the relative abundances of *Bifidobacterium* (reduced), an unclassified genus in the Ruminococcaceae family (reduced), and *Bacteroides* (increased) [53]. The same authors, in other papers, suggest that coadministration of probiotics with an LFD can restore *Bifidobacterium* [52], or that a β-Galactooligosaccharide supplement taken alongside the LFD can improve IBS symptoms [54]. Our results suggest that the LFD nonresponders tended to have reduced α diversity, as measured by the inverse Simpson index, whereas responders did not. *Faecalibacterium prausnitzii*, a commensal bacterium of the Ruminococcaceae family that produces short-chain fatty acids (SCFAs) from dietary fiber [55], was significantly reduced by the LFD and restored by the reintroduction of foods high in FODMAPs. It should be noted in this context that it was *Faecalibacterium prausnitzii* H that was significantly increased; G, E, J, K, and I were decreased, while C and F were increased, but none significantly so (Multimedia Appendix 3). The main explanations for the discrepancies between studies investigating the effects of an LFD on microbiota include, but are not limited to, different DNA extraction protocols [56,57]; different storage conditions [58]; different parameters and bioinformatic analyses, including different comparisons “between groups” rather than changes “within groups”; and use of different sequencing methods [59].

Although significant median increases in symptoms were observed during reintroduction of high-FODMAP foods, in this study, and relative to the changes caused by the LFD, a post hoc analysis (ie, a linear mixed-effect model based on clinical metadata) suggests that sustained long-term symptom control and improvement of QoL could be obtained relative to patients’ baseline. Sustained symptom control during reintroduction has recently been documented by other researchers in Italy, who concluded that the benefit of an LFD persisted during reintroduction (after 3 months) and at 6 months follow-up [60]. Based on this study’s results, the combination of using a food preference approach for reintroduction and eHealth seems fit for long-term disease management. However, further eHealth studies with larger cohorts are needed to confirm these results.

### IBS and Web-Based Management

Other researchers have suggested that web-based and app-based LFD and probiotic treatments may provide therapeutic benefits [61] and should be more widely implemented for IBS management [13]. This study supports these existing results and recommendations and adds further evidence of using eHealth for clinical decision making and long-term management.

However, it is our recommendation that future electronic-based IBS management include valid and quick point-of-care measures that can (1) predict response to treatments and (2) be correlated to symptom severity scores, both of which can support the patients and the personnel doing the daily web-based ward rounds in tailoring treatments and guiding their supervision. We were unable to predict response to treatments (ie, probiotic treatment and LFD) based on the microbiota alone. Others have also failed in this attempt in IBS using a different method (ie, 16S ribosomal RNA [rRNA] next-generation sequencing) than the one we used [53]. It could be that predicting a response to treatment and symptom activity would gain from larger cohorts and more detailed analyses of the gut ecosystem (eg, including other microbes, such as parasites [62]) and metabolites. A promising, low-cost, and noninvasive measure for predicting response to LFD and probiotic treatments in IBS patients is currently under development by researchers at King’s College London that examines volatile organic compounds (VOCs) in feces (ie, VOC profiling) [63]; future evaluation of this method will hopefully determine whether it has clinical utility. A final, important aspect of research into long-term IBS management is the exclusion of other gastrointestinal diseases and comorbidities, as these frequently occur together [44]. A review by Kim et al [64] reported that FC had the highest sensitivity and specificity relative to IBD, whereas fecal SCFAs were most accurate relative to HCs. In the present study, neuroendocrine tumor showed itself with repeatedly elevated FC measures, but this measure did not indicate bile acid malabsorption or microscopic colitis. Future research might reveal new and valid point-of-care markers for detection of other diseases, for surveillance of IBS activity (ie, predictors for symptom relapse), and for predicting responses to therapeutics, thereby helping us tailor treatments and their timing, via improved surveillance, for patients.

### Strengths and Limitations

The strength of this study is its use of an updated and expanded eHealth web application to monitor and treat IBS patients according to their treatment response across 1 year of follow-up. To our knowledge, this is the first eHealth trial to monitor the effect, prospectively and longitudinally, of multiple probiotic treatments and the LFD with subsequent reintroduction of foods high in FODMAPs, and to compare responses to patients’ microbiota.

The main limitations of the study are its sample size and that it is based on a single study center. Another limitation is the use of the Rome III criteria rather than the Rome IV criteria for IBS [65]. Furthermore, gut microbiota were analyzed using fecal samples and metagenomic shotgun sequencing as a proxy. While metagenomic, whole-genome shotgun sequencing allows for a better genome resolution compared to 16S rRNA amplicon sequencing, organisms that are extremely GC-rich or -poor will be underrepresented [66]. Due to costs, samples were also sequenced at a lower depth (an average of 20 M reads), which limits the possibility of detecting microbes in very low abundances. In order to analyze the microbiota, we used a read-based mapping approach that is faster than metagenomic genome assembly, avoids the many problems that can occur during assembly, and provides full-genome information for low-abundance microbes that cannot be assembled otherwise [67]. However, this also meant that we could not capture the specific capabilities of each microbe in a patient, encoded by their particular genome. The *in situ* genome sequences likely differ compared to our reference due to, for example, horizontal gene transfer.

### Conclusions

In conclusion, we have shown the IBS CC web application to be practical, safe, and useful for clinical decision making in the long-term management of IBS. A probiotic treatment, VSL#3, and the LFD were equally efficacious in short- and long-term treatment strategies for managing IBS. We failed to predict response to treatments based on patients’ fecal microbiota, which might someday help individualize treatments. To confirm our results, larger studies are needed and would ideally combine LFD and probiotic treatments or other medical options for managing IBS, especially for nonresponders to the LFD and probiotic treatments.

## Appendix

Multimedia Appendix 1 CONSORT-eHEALTH checklist (V 1.6.1).

Multimedia Appendix 2 Supplementary elaborations, tables, and figures.

Multimedia Appendix 3 Differential species abundances according to responder types.

Multimedia Appendix 4 Differential Kyoto Encyclopedia of Genes and Genomes modules according to responder types.

## Abbreviations

BSC: Bristol Stool Chart
CE: Conformité Européenne
CFU: colony-forming units
FARS: fermentable oligosaccharides, disaccharides, monosaccharides, and polyols (FODMAP) Adherence Report Scale
FC: fecal calprotectin
FODMAP: fermentable oligosaccharides, disaccharides, monosaccharides, and polyols
HC: healthy control
IBD: inflammatory bowel disease
IBS: irritable bowel syndrome
IBS CC: irritable bowel syndrome-constant care
IBS-D: irritable bowel syndrome–diarrhea
IBS-M: irritable bowel syndrome–mixed typed
IBS-QoL: irritable bowel syndrome quality of life
IBS-SSS: Irritable Bowel Syndrome Severity Scoring System
LFD: low-fermentable oligosaccharides, disaccharides, monosaccharides, and polyols (FODMAP) diet
MARS: Medical Adherence Report Scale
NICE: National Institute for Health and Care Excellence
PROM: patient-reported outcome measure
QoL: quality of life
rRNA: ribosomal RNA
SCFA: short-chain fatty acid
subsp: subspecies
UMAP: uniform manifold approximation and projection
VAS: visual analog scale
VOC: volatile organic compound
