# Supplementary material for: Long-Term Effects of a Web-Based Low-FODMAP Diet Versus Probiotic Treatment for Irritable Bowel Syndrome, Including Shotgun Analyses of Microbiota: Randomized, Double-Crossover Clinical Trial
Source: J Med Internet Res. 2021 Dec 14;23(12):e30291. doi: 10.2196/30291 (PMC8715363; doi:10.2196/30291)
Supplement: Multimedia Appendix 1 [file jmir_v23i12e30291_app1.pdf]

CONSORT-EHEALTH (V 1.6.1) - Submission/Publication Form

The CONSORT-EHEALTH checklist is intended for authors of randomized trials evaluating web-based and Internet-based applications/interventions, including mobile interventions, electronic games (incl multiplayer games), social media, certain telehealth applications, and other interactive and/or networked electronic applications. Some of the items (e.g. all subitems under item 5 - description of the intervention) may also be applicable for other study designs.

The goal of the CONSORT EHEALTH checklist and guideline is to be
a) a guide for reporting for authors of RCTs,
b) to form a basis for appraisal of an ehealth trial (in terms of validity)

CONSORT-EHEALTH items/subitems are MANDATORY reporting items for studies published in the Journal of Medical Internet Research and other journals / scientific societies endorsing the checklist.

Items numbered 1., 2., 3., 4a., 4b etc are original CONSORT or CONSORT-NPT (non-pharmacologic treatment) items.
Items with Roman numerals (i., ii, iii, iv etc.) are CONSORT-EHEALTH extensions/clarifications.

As the CONSORT-EHEALTH checklist is still considered in a formative stage, we would ask that you also RATE ON A SCALE OF 1-5 how important/useful you feel each item is FOR THE PURPOSE OF THE CHECKLIST and reporting guideline (optional).

Mandatory reporting items are marked with a red *.
In the textboxes, either copy & paste the relevant sections from your manuscript into this form - please include any quotes from your manuscript in QUOTATION MARKS,
or answer directly by providing additional information not in the manuscript, or elaborating on why the item was not relevant for this study.

YOUR ANSWERS WILL BE PUBLISHED AS A SUPPLEMENTARY FILE TO YOUR PUBLICATION IN JMIR AND ARE CONSIDERED PART OF YOUR PUBLICATION (IF ACCEPTED).
Please fill in these questions diligently. Information will not be copyedited, so please use proper spelling and grammar, use correct capitalization, and avoid abbreviations.

DO NOT FORGET TO SAVE AS PDF _AND_ CLICK THE SUBMIT BUTTON SO YOUR ANSWERS ARE IN OUR DATABASE !!!

Citation Suggestion (if you append the pdf as Appendix we suggest to cite this paper in the caption):
Eysenbach G, CONSORT-EHEALTH Group
CONSORT-EHEALTH: Improving and Standardizing Evaluation Reports of Web-based and Mobile Health Interventions
J Med Internet Res 2011;13(4):e126
URL: http://www.jmir.org/2011/4/e126/

doi: 10.2196/jmir.1923
PMID: 22209829

*Skal udfyldes

Your name *

First Last

Dorit Vedel Ankersen

Primary Affiliation (short), City, Country *

University of Toronto, Toronto, Canada

North Zealand University Hospital, Frederikssu

Your e-mail address *

abc@gmail.com

doritvedel@gmail.com

Title of your manuscript *

Provide the (draft) title of your manuscript.

Long-term effects of a web-based low FODMAP diet vs. probiotic treatment for irritable bowel syndrome, including shotgun analyses of microbiota: a randomized, double crossover clinical trial

Name of your App/Software/Intervention *

If there is a short and a long/alternate name, write the short name first and add the long name in brackets.

IBS CC [IBS Constant Care]

Evaluated Version (if any)

e.g. "V1", "Release 2017-03-01", "Version 2.0.27913"

V2 release 2018-08-23

Language(s) *

What language is the intervention/app in? If multiple languages are available, separate by comma (e.g. "English, French")

Danish

URL of your Intervention Website or App

e.g. a direct link to the mobile app on app in appstore (itunes, Google Play), or URL of the website. If the intervention is a DVD or hardware, you can also link to an Amazon page.

https://ibs.constant-care.com/

URL of an image/screenshot (optional)

Dit svar

Accessibility *

Can an enduser access the intervention presently?

- ( ) access is free and open
- ( ) access only for special usergroups, not open
- ( ) access is open to everyone, but requires payment/subscription/in-app purchases
- (x) app/intervention no longer accessible
- ( ) Andet:

Primary Medical Indication/Disease/Condition *

e.g. "Stress", "Diabetes", or define the target group in brackets after the condition, e.g. "Autism (Parents of children with)", "Alzheimers (Informal Caregivers of)"

Irritable Bowel Syndrome

Primary Outcomes measured in trial *

comma-separated list of primary outcomes reported in the trial

Symptom severity, fecal microbiota

Secondary/other outcomes

Are there any other outcomes the intervention is expected to affect?

Quality of life, sustained symptom control, patient satisfaction with the web-app (IBS CC)

Recommended "Dose" *

What do the instructions for users say on how often the app should be used?

- ○ Approximately Daily
- ◉ Approximately Weekly
- ○ Approximately Monthly
- ○ Approximately Yearly
- ○ "as needed"
- ○ Andet:

Approx. Percentage of Users (starters) still using the app as recommended after 3 months *

- ( ) unknown / not evaluated
- ( ) 0-10%
- ( ) 11-20%
- ( ) 21-30%
- ( ) 31-40%
- ( ) 41-50%
- ( ) 51-60%
- ( ) 61-70%
- ( ) 71%-80%
- ( ) 81-90%
- ( ) 91-100%
- (•) Andet: The web trial has ended, and the app will not be accessible before it h

Overall, was the app/intervention effective? *

- ( ) yes: all primary outcomes were significantly better in intervention group vs control
- ( ) partly: SOME primary outcomes were significantly better in intervention group vs control
- ( ) no statistically significant difference between control and intervention
- ( ) potentially harmful: control was significantly better than intervention in one or more outcomes
- ( ) inconclusive: more research is needed
- (•) Andet: The web-study was not a controlled trial. It compared two web interve

Article Preparation Status/Stage *

At which stage in your article preparation are you currently (at the time you fill in this form)

- ○ not submitted yet - in early draft status
- ◉ not submitted yet - in late draft status, just before submission
- ○ submitted to a journal but not reviewed yet
- ○ submitted to a journal and after receiving initial reviewer comments
- ○ submitted to a journal and accepted, but not published yet
- ○ published
- ○ Andet:

Journal *

If you already know where you will submit this paper (or if it is already submitted), please provide the journal name (if it is not JMIR, provide the journal name under "other")

- ○ not submitted yet / unclear where I will submit this
- ◉ Journal of Medical Internet Research (JMIR)
- ○ JMIR mHealth and UHealth
- ○ JMIR Serious Games
- ○ JMIR Mental Health
- ○ JMIR Public Health
- ○ JMIR Formative Research
- ○ Other JMIR sister journal
- ○ Andet:

Is this a full powered effectiveness trial or a pilot/feasibility trial? *

- (●) Pilot/feasibility
- ( ) Fully powered

Manuscript tracking number *

If this is a JMIR submission, please provide the manuscript tracking number under "other" (The ms tracking number can be found in the submission acknowledgement email, or when you login as author in JMIR. If the paper is already published in JMIR, then the ms tracking number is the four-digit number at the end of the DOI, to be found at the bottom of each published article in JMIR)

- (●) no ms number (yet) / not (yet) submitted to / published in JMIR
- ( ) Andet:

TITLE AND ABSTRACT

1a) TITLE: Identification as a randomized trial in the title

1a) Does your paper address CONSORT item 1a? *

I.e does the title contain the phrase "Randomized Controlled Trial"? (if not, explain the reason under "other")

- ( ) yes
- (●) Andet: The web study was not a controlled trial. It compared two web interve

1a-i) Identify the mode of delivery in the title

Identify the mode of delivery. Preferably use “web-based” and/or “mobile” and/or “electronic game” in the title. Avoid ambiguous terms like “online”, “virtual”, “interactive”. Use “Internet-based” only if Intervention includes non-web-based Internet components (e.g. email), use “computer-based” or “electronic” only if offline products are used. Use “virtual” only in the context of “virtual reality” (3-D worlds). Use “online” only in the context of “online support groups”. Complement or substitute product names with broader terms for the class of products (such as “mobile” or “smart phone” instead of “iphone”), especially if the application runs on different platforms.

	1	2	3	4	5	
subitem not at all important	○	○	○	○	◉	essential

Ryd markering

Does your paper address subitem 1a-i? *

Copy and paste relevant sections from manuscript title (include quotes in quotation marks "like this" to indicate direct quotes from your manuscript), or elaborate on this item by providing additional information not in the ms, or briefly explain why the item is not applicable/relevant for your study

web-based low FODMAP diet vs. probiotic treatment

1a-ii) Non-web-based components or important co-interventions in title

Mention non-web-based components or important co-interventions in title, if any (e.g., “with telephone support”).

	1	2	3	4	5	
subitem not at all important	○	○	◉	○	○	essential

Ryd markering

Does your paper address subitem 1a-ii?

Copy and paste relevant sections from manuscript title (include quotes in quotation marks "like this" to indicate direct quotes from your manuscript), or elaborate on this item by providing additional information not in the ms, or briefly explain why the item is not applicable/relevant for your study

At inclusion in the study, participants were taught how to use the apps (Low FODMAP diet app, CalproSmart app, and IBS CC), dietary education and probiotic treatments were also given to the participants on-site i.e. at the hospital

1a-iii) Primary condition or target group in the title

Mention primary condition or target group in the title, if any (e.g., “for children with Type I Diabetes”) Example: A Web-based and Mobile Intervention with Telephone Support for Children with Type I Diabetes: Randomized Controlled Trial

	1	2	3	4	5	
subitem not at all important	○	○	○	○	◉	essential

Ryd markering

Does your paper address subitem 1a-iii? *

Copy and paste relevant sections from manuscript title (include quotes in quotation marks "like this" to indicate direct quotes from your manuscript), or elaborate on this item by providing additional information not in the ms, or briefly explain why the item is not applicable/relevant for your study

for irritable bowel syndrome

1b) ABSTRACT: Structured summary of trial design, methods, results, and conclusions

NPT extension: Description of experimental treatment, comparator, care providers, centers, and blinding status.

1b-i) Key features/functionalities/components of the intervention and comparator in the METHODS section of the ABSTRACT

Mention key features/functionalities/components of the intervention and comparator in the abstract. If possible, also mention theories and principles used for designing the site. Keep in mind the needs of systematic reviewers and indexers by including important synonyms. (Note: Only report in the abstract what the main paper is reporting. If this information is missing from the main body of text, consider adding it)

	1	2	3	4	5	
subitem not at all important	○	○	◉	○	○	essential

Ryd markering

Does your paper address subitem 1b-i? *

Copy and paste relevant sections from the manuscript abstract (include quotes in quotation marks "like this" to indicate direct quotes from your manuscript), or elaborate on this item by providing additional information not in the ms, or briefly explain why the item is not applicable/relevant for your study

"Adult IBS patients were enrolled in an open-label, randomized crossover trial (for non-responders) with one year of follow-up using the web app, IBS Constant Care (CC)"

1b-ii) Level of human involvement in the METHODS section of the ABSTRACT

Clarify the level of human involvement in the abstract, e.g., use phrases like “fully automated” vs. “therapist/nurse/care provider/physician-assisted” (mention number and expertise of providers involved, if any). (Note: Only report in the abstract what the main paper is reporting. If this information is missing from the main body of text, consider adding it)

	1	2	3	4	5	
subitem not at all important	○	○	○	○	◉	essential

Ryd markering

Does your paper address subitem 1b-ii?

Copy and paste relevant sections from the manuscript abstract (include quotes in quotation marks "like this" to indicate direct quotes from your manuscript), or elaborate on this item by providing additional information not in the ms, or briefly explain why the item is not applicable/relevant for your study

"Web ward rounds were performed daily by the study investigator"

1b-iii) Open vs. closed, web-based (self-assessment) vs. face-to-face assessments in the METHODS section of the ABSTRACT

Mention how participants were recruited (online vs. offline), e.g., from an open access website or from a clinic or a closed online user group (closed usergroup trial), and clarify if this was a purely web-based trial, or there were face-to-face components (as part of the intervention or for assessment). Clearly say if outcomes were self-assessed through questionnaires (as common in web-based trials). Note: In traditional offline trials, an open trial (open-label trial) is a type of clinical trial in which both the researchers and participants know which treatment is being administered. To avoid confusion, use “blinded” or “unblinded” to indicated the level of blinding instead of “open”, as “open” in web-based trials usually refers to “open access” (i.e. participants can self-enrol). (Note: Only report in the abstract what the main paper is reporting. If this information is missing from the main body of text, consider adding it)

	1	2	3	4	5	
subitem not at all important	○	○	○	◉	○	essential

Ryd markering

Does your paper address subitem 1b-iii?

Copy and paste relevant sections from the manuscript abstract (include quotes in quotation marks "like this" to indicate direct quotes from your manuscript), or elaborate on this item by providing additional information not in the ms, or briefly explain why the item is not applicable/relevant for your study

"This eHealth study was an open-label, randomized, crossover trial (for non-responders), with one year of follow-up (Figure 1)......Patients were recruited from the outpatient clinic at the Department of Gastroenterology, North Zealand University Hospital, Denmark."

1b-iv) RESULTS section in abstract must contain use data

Report number of participants enrolled/assessed in each group, the use/uptake of the intervention (e.g., attrition/adherence metrics, use over time, number of logins etc.), in addition to primary/secondary outcomes. (Note: Only report in the abstract what the main paper is reporting. If this information is missing from the main body of text, consider adding it)

	1	2	3	4	5	
subitem not at all important	○	○	○	◉	○	essential

Ryd markering

Does your paper address subitem 1b-iv?

Copy and paste relevant sections from the manuscript abstract (include quotes in quotation marks "like this" to indicate direct quotes from your manuscript), or elaborate on this item by providing additional information not in the ms, or briefly explain why the item is not applicable/relevant for your study

"A total of 34 IBS patients without co-morbidities and six healthy controls were enrolled in the study. Taken from participating subjects, 180 fecal samples were analyzed for their microbiota composition. Twelve IBS patients responded to LFD (57%) and eight completed the reintroduction of FODMAPs (38%). Thirteen (62%) patients responded to their first treatment of VSL#3®, and seven (33%) responded to multiple VSL#3® treatments.
No significant difference in the median reduction of IBS-SSS for LFD vs. probiotic responders was observed, where for LFD it was -126.50 (IQR: -196.75; -76.75) and for VSL#3® it was -130.00 (IQR: -211.00; -70.50), P=1. Responses to either treatment were not able to be predicted using patients' microbiota."

1b-v) CONCLUSIONS/DISCUSSION in abstract for negative trials

Conclusions/Discussions in abstract for negative trials: Discuss the primary outcome - if the trial is negative (primary outcome not changed), and the intervention was not used, discuss whether negative results are attributable to lack of uptake and discuss reasons. (Note: Only report in the abstract what the main paper is reporting. If this information is missing from the main body of text, consider adding it)

	1	2	3	4	5	
subitem not at all important	○	○	◉	○	○	essential

Ryd markering

Does your paper address subitem 1b-v?

Copy and paste relevant sections from the manuscript abstract (include quotes in quotation marks "like this" to indicate direct quotes from your manuscript), or elaborate on this item by providing additional information not in the ms, or briefly explain why the item is not applicable/relevant for your study

"No significant difference in the median reduction of IBS-SSS for LFD vs. probiotic responders was observed, where for LFD it was -126.50 (IQR: -196.75; -76.75) and for VSL#3® it was -130.00 (IQR: -211.00; -70.50), P=1. Responses to either treatment were not able to be predicted using patients' microbiota."

INTRODUCTION

2a) In INTRODUCTION: Scientific background and explanation of rationale

2a-i) Problem and the type of system/solution

Describe the problem and the type of system/solution that is object of the study: intended as stand-alone intervention vs. incorporated in broader health care program? Intended for a particular patient population? Goals of the intervention, e.g., being more cost-effective to other interventions, replace or complement other solutions? (Note: Details about the intervention are provided in “Methods” under 5)

	1	2	3	4	5	
subitem not at all important	○	○	○	○	◉	essential

Ryd markering

Does your paper address subitem 2a-i? *

Copy and paste relevant sections from the manuscript (include quotes in quotation marks "like this" to indicate direct quotes from your manuscript), or elaborate on this item by providing additional information not in the ms, or briefly explain why the item is not applicable/relevant for your study

"Managing IBS continues to be challenging because of the complexity of its chronicity, heterogeneous patient groups with and without co-morbidities, and a lack of both diagnostic tools and of well-documented treatment strategies for long-term purposes. Treatment strategies for IBS range from lifestyle and dietary advice to pharmacological solutions that generally target only the primary symptom. Recently, holistic eHealth solutions that monitor symptoms and support patients with digestives diseases have proven valuable in involving and empowering patients, and are used as an adjuvant to current treatments regimes [7,8]."

2a-ii) Scientific background, rationale: What is known about the (type of) system

Scientific background, rationale: What is known about the (type of) system that is the object of the study (be sure to discuss the use of similar systems for other conditions/diagnoses, if appropiate), motivation for the study, i.e. what are the reasons for and what is the context for this specific study, from which stakeholder viewpoint is the study performed, potential impact of findings [2]. Briefly justify the choice of the comparator.

	1	2	3	4	5	
subitem not at all important	○	○	○	◉	○	essential

Ryd markering

Does your paper address subitem 2a-ii? *

Copy and paste relevant sections from the manuscript (include quotes in quotation marks "like this" to indicate direct quotes from your manuscript), or elaborate on this item by providing additional information not in the ms, or briefly explain why the item is not applicable/relevant for your study

"A previous study has shown that short-term, web-based management of IBS patients, while treating their symptoms with LFD and probiotics, is practical and effective [13]. Yet the LFD has also been linked to a reduction in beneficial gastrointestinal bacteria [14] and to compromise nutritional intake, especially calcium [15].....So far, no one has been able to fingerprint patients based on their fecal microbial composition and tailor a particular intervention for them individually [4]."

2b) In INTRODUCTION: Specific objectives or hypotheses

Does your paper address CONSORT subitem 2b? *

Copy and paste relevant sections from the manuscript (include quotes in quotation marks "like this" to indicate direct quotes from your manuscript), or elaborate on this item by providing additional information not in the ms, or briefly explain why the item is not applicable/relevant for your study

"The primary aim of this one-year, web-based study was to determine if a four-week probiotic treatment (VSL#3®) and LFD were equally as effective at reducing IBS symptoms and whether the response to either of the two treatments could be explained by fecal microbiota. The study's secondary aims were to evaluate patients' quality of life, the effect of multiple VSL#3® treatments in sustaining symptom control across one year, and reintroduction of FODMAPs among LFD responders, as well as to evaluate the web application, ibs.constant-care.com (IBS CC), for its efficacy in long-term clinical use."

METHODS

3a) Description of trial design (such as parallel, factorial) including allocation ratio

Does your paper address CONSORT subitem 3a? *

Copy and paste relevant sections from the manuscript (include quotes in quotation marks "like this" to indicate direct quotes from your manuscript), or elaborate on this item by providing additional information not in the ms, or briefly explain why the item is not applicable/relevant for your study

"This eHealth study was an open-label, randomized, crossover trial (for non-responders), with one year of follow-up (Figure 1)....Figure 1. One-year study design. IBS Constant Care web app (IBS CC) was used for home-monitoring of symptoms and clinical decision-making. Treatments: Four weeks of monitoring on IBS CC, followed by randomization to either a four-week low FODMAP diet (LFD) or probiotic treatment (VSL#3®, Actial Pharma). Responders (R) to LFD would subsequently be reintroduced to foods higher in fermentable oligo-, di-, monosaccharides and polyols (FODMAPs). Responders to probiotic treatment would receive multiple treatments upon symptom flare-ups (an increase in IBS severity scoring system, IBS-SSS, of more than 50 points)."

3b) Important changes to methods after trial commencement (such as eligibility criteria), with reasons

Does your paper address CONSORT subitem 3b? *

Copy and paste relevant sections from the manuscript (include quotes in quotation marks "like this" to indicate direct quotes from your manuscript), or elaborate on this item by providing additional information not in the ms, or briefly explain why the item is not applicable/relevant for your study

Yes, changes were made to the inclusion criteria of IBS patients and the method used to analyze the microbiota. Important changes made in the study protocol: BMI [18.5-25] --> [18.5-35] and 16S rRNA --> shotgun metagenomic analysis. All changes have been approved by the Ethical Committee of Denmark.

3b-i) Bug fixes, Downtimes, Content Changes

Bug fixes, Downtimes, Content Changes: ehealth systems are often dynamic systems. A description of changes to methods therefore also includes important changes made on the intervention or comparator during the trial (e.g., major bug fixes or changes in the functionality or content) (5-iii) and other “unexpected events” that may have influenced study design such as staff changes, system failures/downtimes, etc. [2].

	1	2	3	4	5	
subitem not at all important	○	○	◉	○	○	essential

Ryd markering

Does your paper address subitem 3b-i?

Copy and paste relevant sections from the manuscript (include quotes in quotation marks "like this" to indicate direct quotes from your manuscript), or elaborate on this item by providing additional information not in the ms, or briefly explain why the item is not applicable/relevant for your study

Content remained the same during the one-year intervention. Unfortunately, 'time-outs' were experienced by some end-users during the study course (addressed in the supplementary material=Multimedia Appendix 2)

4a) Eligibility criteria for participants

Does your paper address CONSORT subitem 4a? *

Copy and paste relevant sections from the manuscript (include quotes in quotation marks "like this" to indicate direct quotes from your manuscript), or elaborate on this item by providing additional information not in the ms, or briefly explain why the item is not applicable/relevant for your study

"Adult IBS patients (18 years or older) fulfilling the Rome III criteria and sub-diagnosed with either IBS-Diarrhea (IBS-D) or IBS-Mixed type (IBS-M) by a gastroenterologist were consecutively included at North Zealand University Hospital (August 23, 2018 to October 18, 2019). Patients were excluded if they did not have a smartphone; had previously undergone gastrointestinal surgery; had been diagnosed with celiac disease or lactose intolerance; had been sub-diagnosed with constipation (IBS-C) or unspecified IBS (IBS-U); were pregnant; followed alternative diets; had a history of alcohol or drug abuse; had a BMI below 18.5 or above 35; had been diagnosed with any co-morbidities, e.g., diabetes; had previously been on a low FODMAP diet guided by a dietician; had been on any probiotic or antibiotic treatment within the three months prior to inclusion; or had an IBS severity score lower than 175 at inclusion.
Healthy controls (HCs) older than 18 years, with a normal BMI (18.5-25), fecal calprotectin (FC) less than 70 mg/kg, and not taking daily medication nor food supplements were recruited internally from our institution. "

4a-i) Computer / Internet literacy

Computer / Internet literacy is often an implicit “de facto” eligibility criterion - this should be explicitly clarified.

	1	2	3	4	5	
subitem not at all important	○	○	○	○	◉	essential

Ryd markering

Does your paper address subitem 4a-i?

Copy and paste relevant sections from the manuscript (include quotes in quotation marks "like this" to indicate direct quotes from your manuscript), or elaborate on this item by providing additional information not in the ms, or briefly explain why the item is not applicable/relevant for your study

"Patients were excluded if they did not have a smartphone....."

4a-ii) Open vs. closed, web-based vs. face-to-face assessments:

Open vs. closed, web-based vs. face-to-face assessments: Mention how participants were recruited (online vs. offline), e.g., from an open access website or from a clinic, and clarify if this was a purely web-based trial, or there were face-to-face components (as part of the intervention or for assessment), i.e., to what degree got the study team to know the participant. In online-only trials, clarify if participants were quasi-anonymous and whether having multiple identities was possible or whether technical or logistical measures (e.g., cookies, email confirmation, phone calls) were used to detect/prevent these.

	1	2	3	4	5	
subitem not at all important	○	○	○	○	◉	essential

Ryd markering

Does your paper address subitem 4a-ii? *

Copy and paste relevant sections from the manuscript (include quotes in quotation marks "like this" to indicate direct quotes from your manuscript), or elaborate on this item by providing additional information not in the ms, or briefly explain why the item is not applicable/relevant for your study

"IBS patients were consecutively included at North Zealand University Hospital (August 23, 2018 to October 18, 2019)...This eHealth study was an open-label, randomized, crossover trial (for non-responders), with one year of follow-up (Figure 1)." Inclusion in the study, how to use the apps (Low FODMAP diet app, CalproSmart app, and IBS CC), dietary education and probiotic treatments were given to the patients on-site i.e. at the hospital (face to face).

4a-iii) Information giving during recruitment

Information given during recruitment. Specify how participants were briefed for recruitment and in the informed consent procedures (e.g., publish the informed consent documentation as appendix, see also item X26), as this information may have an effect on user self-selection, user expectation and may also bias results.

	1	2	3	4	5	
subitem not at all important	○	○	○	○	◉	essential

Ryd markering

Does your paper address subitem 4a-iii?

Copy and paste relevant sections from the manuscript (include quotes in quotation marks "like this" to indicate direct quotes from your manuscript), or elaborate on this item by providing additional information not in the ms, or briefly explain why the item is not applicable/relevant for your study

"Participants were recruited and briefed by MDs and study investigator from the outpatient clinic, North Zealand University, Denmark. All information to the participants was approved by the Ethical Committee of Denmark (H-16023499). All patients in the study gave written informed consent prior to their inclusion."

4b) Settings and locations where the data were collected

Does your paper address CONSORT subitem 4b? *

Copy and paste relevant sections from the manuscript (include quotes in quotation marks "like this" to indicate direct quotes from your manuscript), or elaborate on this item by providing additional information not in the ms, or briefly explain why the item is not applicable/relevant for your study

Clinical metadata was exported from the IBS CC database for statistical analyses.

4b-i) Report if outcomes were (self-)assessed through online questionnaires

Clearly report if outcomes were (self-)assessed through online questionnaires (as common in web-based trials) or otherwise.

	1	2	3	4	5	
subitem not at all important	○	○	○	○	◉	essential

Ryd markering

Does your paper address subitem 4b-i? *

Copy and paste relevant sections from the manuscript (include quotes in quotation marks "like this" to indicate direct quotes from your manuscript), or elaborate on this item by providing additional information not in the ms, or briefly explain why the item is not applicable/relevant for your study

Self-assessed patient-reported outcome measures (PROMs) included in the IBS CC are described in the method section in the manuscript

4b-ii) Report how institutional affiliations are displayed

Report how institutional affiliations are displayed to potential participants [on ehealth media], as affiliations with prestigious hospitals or universities may affect volunteer rates, use, and reactions with regards to an intervention.(Not a required item – describe only if this may bias results)

	1	2	3	4	5	
subitem not at all important	○	○	○	○	◉	essential

Ryd markering

Does your paper address subitem 4b-ii?

Copy and paste relevant sections from the manuscript (include quotes in quotation marks "like this" to indicate direct quotes from your manuscript), or elaborate on this item by providing additional information not in the ms, or briefly explain why the item is not applicable/relevant for your study

Institutional affiliation is displayed to potential participants in the information material (approved by the ethical committee of Denmark) and as well on the web-app IBS CC.

5) The interventions for each group with sufficient details to allow replication, including how and when they were actually administered

5-i) Mention names, credential, affiliations of the developers, sponsors, and owners

Mention names, credential, affiliations of the developers, sponsors, and owners [6] (if authors/evaluators are owners or developer of the software, this needs to be declared in a “Conflict of interest” section or mentioned elsewhere in the manuscript).

	1	2	3	4	5	
subitem not at all important	○	○	○	○	◉	essential

Ryd markering

Does your paper address subitem 5-i?

Copy and paste relevant sections from the manuscript (include quotes in quotation marks "like this" to indicate direct quotes from your manuscript), or elaborate on this item by providing additional information not in the ms, or briefly explain why the item is not applicable/relevant for your study

"Grants supporting this study were provided by North Zealand University Hospital, Ferring Pharmaceuticals, Handelsgartner Ove William Buhl Olesen and ægtefælle fru Edith Buhl Olesens memorial fund, Dagmar and Ivar Enevoldsen memorial fund and the Crohn’s & Colitis Patient Society, Denmark. This study was also supported in part by Unseen Bio Aps and Calpro AS."

"We wish to thank Thomas Janum (J-Consult) for support in developing the IBS Constant Care application, integration of CalproSmartTM, and the export function from the Constant Care database."

5-ii) Describe the history/development process

Describe the history/development process of the application and previous formative evaluations (e.g., focus groups, usability testing), as these will have an impact on adoption/use rates and help with interpreting results.

	1	2	3	4	5	
subitem not at all important	○	○	◉	○	○	essential

Ryd markering

Does your paper address subitem 5-ii?

Copy and paste relevant sections from the manuscript (include quotes in quotation marks "like this" to indicate direct quotes from your manuscript), or elaborate on this item by providing additional information not in the ms, or briefly explain why the item is not applicable/relevant for your study

"IBS CC has previously been used in other web-based studies [24,25]. The updated and expanded version of IBS CC (version 2.0) used in this study includes various patient-reported outcome measures (PROMs), listed in Multimedia Appendix 2 and described in detail below. Patient-reported satisfaction with the current version of IBS CC was evaluated before randomization and at one-year-follow up. IBS CC (v 2.0) meets the requirements of the EU's General Data Protection Regulation (GDPR)."

5-iii) Revisions and updating

Revisions and updating. Clearly mention the date and/or version number of the application/intervention (and comparator, if applicable) evaluated, or describe whether the intervention underwent major changes during the evaluation process, or whether the development and/or content was "frozen" during the trial. Describe dynamic components such as news feeds or changing content which may have an impact on the replicability of the intervention (for unexpected events see item 3b).

	1	2	3	4	5	
subitem not at all important	○	◉	○	○	○	essential

Ryd markering

Does your paper address subitem 5-iii?

Copy and paste relevant sections from the manuscript (include quotes in quotation marks "like this" to indicate direct quotes from your manuscript), or elaborate on this item by providing additional information not in the ms, or briefly explain why the item is not applicable/relevant for your study

No updates were made during the course of the study

5-iv) Quality assurance methods

Provide information on quality assurance methods to ensure accuracy and quality of information provided [1], if applicable.

	1	2	3	4	5	
subitem not at all important	○	○	◉	○	○	essential

Ryd markering

Does your paper address subitem 5-iv?

Copy and paste relevant sections from the manuscript (include quotes in quotation marks "like this" to indicate direct quotes from your manuscript), or elaborate on this item by providing additional information not in the ms, or briefly explain why the item is not applicable/relevant for your study

Validated PROMs were used when available. These measures are described in the method section of the manuscript.

5-v) Ensure replicability by publishing the source code, and/or providing screenshots/screen-capture video, and/or providing flowcharts of the algorithms used

Ensure replicability by publishing the source code, and/or providing screenshots/screen-capture video, and/or providing flowcharts of the algorithms used. Replicability (i.e., other researchers should in principle be able to replicate the study) is a hallmark of scientific reporting.

	1	2	3	4	5	
subitem not at all important	○	○	◉	○	○	essential

Ryd markering

Does your paper address subitem 5-v?

Copy and paste relevant sections from the manuscript (include quotes in quotation marks "like this" to indicate direct quotes from your manuscript), or elaborate on this item by providing additional information not in the ms, or briefly explain why the item is not applicable/relevant for your study

One-year disease courses of a low FODMAP diet and a probiotic responder are shown in the supplementary material (Multimedia Appendix 2). Published with permission from the patients.

5-vi) Digital preservation

Digital preservation: Provide the URL of the application, but as the intervention is likely to change or disappear over the course of the years; also make sure the intervention is archived (Internet Archive, webcitation.org, and/or publishing the source code or screenshots/videos alongside the article). As pages behind login screens cannot be archived, consider creating demo pages which are accessible without login.

	1	2	3	4	5	
subitem not at all important	○	○	◉	○	○	essential

Ryd markering

Does your paper address subitem 5-vi?

Copy and paste relevant sections from the manuscript (include quotes in quotation marks "like this" to indicate direct quotes from your manuscript), or elaborate on this item by providing additional information not in the ms, or briefly explain why the item is not applicable/relevant for your study

https://ibs.constant-care.com/ (the web-app is, however, no longer active. Might be activated when CE-marked according to MDR)

5-vii) Access

Access: Describe how participants accessed the application, in what setting/context, if they had to pay (or were paid) or not, whether they had to be a member of specific group. If known, describe how participants obtained "access to the platform and Internet" [1]. To ensure access for editors/reviewers/readers, consider to provide a "backdoor" login account or demo mode for reviewers/readers to explore the application (also important for archiving purposes, see vi).

	1	2	3	4	5	
subitem not at all important	○	○	◉	○	○	essential

Ryd markering

Does your paper address subitem 5-vii? *

Copy and paste relevant sections from the manuscript (include quotes in quotation marks "like this" to indicate direct quotes from your manuscript), or elaborate on this item by providing additional information not in the ms, or briefly explain why the item is not applicable/relevant for your study

IBS CC (v 2.0) meets the requirements of the EU's General Data Protection Regulation (GDPR). (IBS CC includes two-factor-authentication).

5-viii) Mode of delivery, features/functionalities/components of the intervention and comparator, and the theoretical framework

Describe mode of delivery, features/functionalities/components of the intervention and comparator, and the theoretical framework [6] used to design them (instructional strategy [1], behaviour change techniques, persuasive features, etc., see e.g., [7, 8] for terminology). This includes an in-depth description of the content (including where it is coming from and who developed it) [1]," whether [and how] it is tailored to individual circumstances and allows users to track their progress and receive feedback" [6]. This also includes a description of communication delivery channels and – if computer-mediated communication is a component – whether communication was synchronous or asynchronous [6]. It also includes information on presentation strategies [1], including page design principles, average amount of text on pages, presence of hyperlinks to other resources, etc. [1].

	1	2	3	4	5	
subitem not at all important	○	○	◉	○	○	essential

Ryd markering

Does your paper address subitem 5-viii? *

Copy and paste relevant sections from the manuscript (include quotes in quotation marks "like this" to indicate direct quotes from your manuscript), or elaborate on this item by providing additional information not in the ms, or briefly explain why the item is not applicable/relevant for your study

All participants had access to IBS CC. This study is not a controlled trial.

5-ix) Describe use parameters

Describe use parameters (e.g., intended “doses” and optimal timing for use). Clarify what instructions or recommendations were given to the user, e.g., regarding timing, frequency, heaviness of use, if any, or was the intervention used ad libitum.

	1	2	3	4	5	
subitem not at all important	○	○	○	◉	○	essential

Ryd markering

Does your paper address subitem 5-ix?

Copy and paste relevant sections from the manuscript (include quotes in quotation marks "like this" to indicate direct quotes from your manuscript), or elaborate on this item by providing additional information not in the ms, or briefly explain why the item is not applicable/relevant for your study

"Instructions to patients of how often they should monitor themselves at home using the web application, IBS CC are listed in Multimedia Appendix 2."

5-x) Clarify the level of human involvement

Clarify the level of human involvement (care providers or health professionals, also technical assistance) in the e-intervention or as co-intervention (detail number and expertise of professionals involved, if any, as well as “type of assistance offered, the timing and frequency of the support, how it is initiated, and the medium by which the assistance is delivered”. It may be necessary to distinguish between the level of human involvement required for the trial, and the level of human involvement required for a routine application outside of a RCT setting (discuss under item 21 – generalizability).

	1	2	3	4	5	
subitem not at all important	○	○	○	◉	○	essential

Ryd markering

Does your paper address subitem 5-x?

Copy and paste relevant sections from the manuscript (include quotes in quotation marks "like this" to indicate direct quotes from your manuscript), or elaborate on this item by providing additional information not in the ms, or briefly explain why the item is not applicable/relevant for your study

"At inclusion, patients were trained (for approximately one hour) in home monitoring of symptoms using IBS CC, including inflammation (measured via FC). FC was measured using the CalproSmartTM app. Patients were instructed to home-monitor every week for at least four weeks before randomization to either the LFD or probiotic treatment......"

"Web ward rounds were performed daily by the study investigator, based on an electronic patient list (green: inactive, yellow: mild to moderate, or red: severe symptoms, according to IBS-SSS)"

5-xi) Report any prompts/reminders used

Report any prompts/reminders used: Clarify if there were prompts (letters, emails, phone calls, SMS) to use the application, what triggered them, frequency etc. It may be necessary to distinguish between the level of prompts/reminders required for the trial, and the level of prompts/reminders for a routine application outside of a RCT setting (discuss under item 21 – generalizability).

	1	2	3	4	5	
subitem not at all important	○	○	◉	○	○	essential

Ryd markering

Does your paper address subitem 5-xi? *

Copy and paste relevant sections from the manuscript (include quotes in quotation marks "like this" to indicate direct quotes from your manuscript), or elaborate on this item by providing additional information not in the ms, or briefly explain why the item is not applicable/relevant for your study

"Instructions to patients of how often they should monitor themselves at home using the web application, IBS CC are listed in Multimedia Appendix 2 and eMail reminders 'time to home-monitor' were sent to participants that accepted this."

5-xii) Describe any co-interventions (incl. training/support)

Describe any co-interventions (incl. training/support): Clearly state any interventions that are provided in addition to the targeted eHealth intervention, as ehealth intervention may not be designed as stand-alone intervention. This includes training sessions and support [1]. It may be necessary to distinguish between the level of training required for the trial, and the level of training for a routine application outside of a RCT setting (discuss under item 21 – generalizability.

	1	2	3	4	5	
subitem not at all important	○	○	◉	○	○	essential

Ryd markering

Does your paper address subitem 5-xii? *

Copy and paste relevant sections from the manuscript (include quotes in quotation marks "like this" to indicate direct quotes from your manuscript), or elaborate on this item by providing additional information not in the ms, or briefly explain why the item is not applicable/relevant for your study

"Patients randomized to the LFD had a consultation at the hospital with a nutritionist, who guided the patients individually through the diet's principles and how to monitor their symptoms (see Multimedia Appendix 2 for home monitoring/screening intervals). Patients randomized to the probiotic treatment were instructed in how to consume the sachets and home-monitor using IBS CC during treatment (see Multimedia Appendix 2)."

6a) Completely defined pre-specified primary and secondary outcome measures, including how and when they were assessed

Does your paper address CONSORT subitem 6a? *

Copy and paste relevant sections from the manuscript (include quotes in quotation marks "like this" to indicate direct quotes from your manuscript), or elaborate on this item by providing additional information not in the ms, or briefly explain why the item is not applicable/relevant for your study

"The primary aim of this one-year, web-based study was to determine if a four-week probiotic treatment (VSL#3®) and LFD were equally as effective at reducing IBS symptoms and whether the response to either of the two treatments could be explained by fecal microbiota......Response was defined as a decrease in IBS-SSS of at least 50 points....Fecal microbiota were analyzed by shotgun metagenomic sequencing (at least 10 million 2x100 bp paired-end sequencing reads per sample)."

6a-i) Online questionnaires: describe if they were validated for online use and apply CHERRIES items to describe how the questionnaires were designed/deployed

If outcomes were obtained through online questionnaires, describe if they were validated for online use and apply CHERRIES items to describe how the questionnaires were designed/deployed [9].

	1	2	3	4	5	
subitem not at all important	○	○	◉	○	○	essential

Ryd markering

Does your paper address subitem 6a-i?

Copy and paste relevant sections from manuscript text

Validated PROMs were used when available, however, validation for online use was not conducted

6a-ii) Describe whether and how "use" (including intensity of use/dosage) was defined/measured/monitored

Describe whether and how "use" (including intensity of use/dosage) was defined/measured/monitored (logins, logfile analysis, etc.). Use/adoption metrics are important process outcomes that should be reported in any ehealth trial.

	1	2	3	4	5	
subitem not at all important	○	○	◉	○	○	essential

Ryd markering

Does your paper address subitem 6a-ii?

Copy and paste relevant sections from manuscript text

Entries were not counted, however, web-consultations and text messages via IBS CC were counted. "During the one-year study period, originating from both eCare provider and participants (n=33, including HCs), 887 web consultations and 781 text messages were registered."

6a-iii) Describe whether, how, and when qualitative feedback from participants was obtained

Describe whether, how, and when qualitative feedback from participants was obtained (e.g., through emails, feedback forms, interviews, focus groups).

	1	2	3	4	5	
subitem not at all important	○	○	○	○	◉	essential

Ryd markering

Does your paper address subitem 6a-iii?

Copy and paste relevant sections from manuscript text

"Participants evaluated their satisfaction with IBS CC, including FC home testing, before their randomization on a visual analogue scale (VAS) from 1-10, where 10 was the greatest possible satisfaction. One-year patient satisfaction with the study and IBS CC, including FC home testing, was assessed using an e-questionnaire in IBS CC and prepared by the authors, consisting of nine yes/no questions and two other questions, one regarding the time used for home monitoring and the other asking for suggestions on improving IBS CC for future clinical use."

6b) Any changes to trial outcomes after the trial commenced, with reasons

Does your paper address CONSORT subitem 6b? *

Copy and paste relevant sections from the manuscript (include quotes in quotation marks "like this" to indicate direct quotes from your manuscript), or elaborate on this item by providing additional information not in the ms, or briefly explain why the item is not applicable/relevant for your study

No changes to outcomes after the trial commenced

7a) How sample size was determined

NPT: When applicable, details of whether and how the clustering by care provides or centers was addressed

7a-i) Describe whether and how expected attrition was taken into account when calculating the sample size

Describe whether and how expected attrition was taken into account when calculating the sample size.

	1	2	3	4	5	
subitem not at all important	○	○	○	●	○	essential

Ryd markering

Does your paper address subitem 7a-i?

Copy and paste relevant sections from manuscript title (include quotes in quotation marks "like this" to indicate direct quotes from your manuscript), or elaborate on this item by providing additional information not in the ms, or briefly explain why the item is not applicable/relevant for your study

"Although the prevalence of IBS is high in westernized countries [1] such as Denmark, we were only able to include 34 out of an estimated target of 104 patients. This was mainly due to a high prevalence of comorbidities among eligible subjects that resulted in our excluding 64% of them....Our aim in excluding participants with multiple conditions, including other medical treatments, was to collect untarnished data for microbial analyses"

7b) When applicable, explanation of any interim analyses and stopping guidelines

Does your paper address CONSORT subitem 7b? *

Copy and paste relevant sections from the manuscript (include quotes in quotation marks "like this" to indicate direct quotes from your manuscript), or elaborate on this item by providing additional information not in the ms, or briefly explain why the item is not applicable/relevant for your study

Not applicable for this study

8a) Method used to generate the random allocation sequence

NPT: When applicable, how care providers were allocated to each trial group

Does your paper address CONSORT subitem 8a? *

Copy and paste relevant sections from the manuscript (include quotes in quotation marks "like this" to indicate direct quotes from your manuscript), or elaborate on this item by providing additional information not in the ms, or briefly explain why the item is not applicable/relevant for your study

online randomization program

8b) Type of randomisation; details of any restriction (such as blocking and block size)

Does your paper address CONSORT subitem 8b? *

Copy and paste relevant sections from the manuscript (include quotes in quotation marks "like this" to indicate direct quotes from your manuscript), or elaborate on this item by providing additional information not in the ms, or briefly explain why the item is not applicable/relevant for your study

"This eHealth study was an open-label, randomized, crossover trial (for non-responders), with one year of follow-up (Figure 1)"

9) Mechanism used to implement the random allocation sequence (such as sequentially numbered containers), describing any steps taken to conceal the sequence until interventions were assigned

Does your paper address CONSORT subitem 9? *

Copy and paste relevant sections from the manuscript (include quotes in quotation marks "like this" to indicate direct quotes from your manuscript), or elaborate on this item by providing additional information not in the ms, or briefly explain why the item is not applicable/relevant for your study

"Figure 1. One-year study design. IBS Constant Care web app (IBS CC) was used for home-monitoring of symptoms and clinical decision-making. Treatments: Four weeks of monitoring on IBS CC, followed by randomization to either a four-week low FODMAP diet (LFD) or probiotic treatment (VSL#3®, Actial Pharma). Responders (R) to LFD would subsequently be reintroduced to foods higher in fermentable oligo-, di-, monosaccharides and polyols (FODMAPs). Responders to probiotic treatment would receive multiple treatments upon symptom flare-ups (an increase in IBS severity scoring system, IBS-SSS, of more than 50 points). Response was defined as a decrease in IBS-SSS of at least 50 points. Non-responders (NR) waited for a minimum of two weeks wash-out before being crossed over (this is not shown in the figure)."

10) Who generated the random allocation sequence, who enrolled participants, and who assigned participants to interventions

Does your paper address CONSORT subitem 10? *

Copy and paste relevant sections from the manuscript (include quotes in quotation marks "like this" to indicate direct quotes from your manuscript), or elaborate on this item by providing additional information not in the ms, or briefly explain why the item is not applicable/relevant for your study

The randomization was blinded to the study investigator.

11a) If done, who was blinded after assignment to interventions (for example, participants, care providers, those assessing outcomes) and how

NPT: Whether or not administering co-interventions were blinded to group assignment

11a-i) Specify who was blinded, and who wasn’t

Specify who was blinded, and who wasn’t. Usually, in web-based trials it is not possible to blind the participants [1, 3] (this should be clearly acknowledged), but it may be possible to blind outcome assessors, those doing data analysis or those administering co-interventions (if any).

	1	2	3	4	5	
subitem not at all important	(•)	( )	( )	( )	( )	essential

Ryd markering

Does your paper address subitem 11a-i? *

Copy and paste relevant sections from the manuscript (include quotes in quotation marks "like this" to indicate direct quotes from your manuscript), or elaborate on this item by providing additional information not in the ms, or briefly explain why the item is not applicable/relevant for your study

Researchers and participants were both unblinded to the intervention type

11a-ii) Discuss e.g., whether participants knew which intervention was the “intervention of interest” and which one was the “comparator”

Informed consent procedures (4a-ii) can create biases and certain expectations - discuss e.g., whether participants knew which intervention was the “intervention of interest” and which one was the “comparator”.

	1	2	3	4	5	
subitem not at all important	(•)	( )	( )	( )	( )	essential

Ryd markering

Does your paper address subitem 11a-ii?

Copy and paste relevant sections from the manuscript (include quotes in quotation marks "like this" to indicate direct quotes from your manuscript), or elaborate on this item by providing additional information not in the ms, or briefly explain why the item is not applicable/relevant for your study

All participants were using the web-app (IBS CC) and both diet and probiotic intervention were of interest.

11b) If relevant, description of the similarity of interventions

(this item is usually not relevant for ehealth trials as it refers to similarity of a placebo or sham intervention to a active medication/intervention)

Does your paper address CONSORT subitem 11b? *

Copy and paste relevant sections from the manuscript (include quotes in quotation marks "like this" to indicate direct quotes from your manuscript), or elaborate on this item by providing additional information not in the ms, or briefly explain why the item is not applicable/relevant for your study

"Figure 1. One-year study design. IBS Constant Care web app (IBS CC) was used for home-monitoring of symptoms and clinical decision-making. Treatments: Four weeks of monitoring on IBS CC, followed by randomization to either a four-week low FODMAP diet (LFD) or probiotic treatment (VSL#3®, Actial Pharma). Responders (R) to LFD would subsequently be reintroduced to foods higher in fermentable oligo-, di-, monosaccharides and polyols (FODMAPs). Responders to probiotic treatment would receive multiple treatments upon symptom flare-ups (an increase in IBS severity scoring system, IBS-SSS, of more than 50 points). Response was defined as a decrease in IBS-SSS of at least 50 points. Non-responders (NR) waited for a minimum of two weeks wash-out before being crossed over (this is not shown in the figure)"

12a) Statistical methods used to compare groups for primary and secondary outcomes

NPT: When applicable, details of whether and how the clustering by care providers or centers was addressed

Does your paper address CONSORT subitem 12a? *

Copy and paste relevant sections from the manuscript (include quotes in quotation marks "like this" to indicate direct quotes from your manuscript), or elaborate on this item by providing additional information not in the ms, or briefly explain why the item is not applicable/relevant for your study

"Counts, means, medians, and interquartile ranges (IQR) were computed from the PROMs. Wilcoxon rank- sum tests or Dunn's all-pair tests were performed to compare medians....A P value smaller than .05 was considered significant. We used a uniform manifold approximation and projection [41] (UMAP) technique in order to visually inspect potential clustering of the Bray-Curtis dissimilarity reduced to two dimensions. We estimated differential sequence abundance using a beta-binomial model provided by the R package, corncob [42], with FDR (<0.1)-adjusted P-values. Clinical outcomes were assessed in a post hoc analysis by linear mixed effect models [43]."

12a-i) Imputation techniques to deal with attrition / missing values

Imputation techniques to deal with attrition / missing values: Not all participants will use the intervention/comparator as intended and attrition is typically high in ehealth trials. Specify how participants who did not use the application or dropped out from the trial were treated in the statistical analysis (a complete case analysis is strongly discouraged, and simple imputation techniques such as LOCF may also be problematic [4]).

	1	2	3	4	5	
subitem not at all important	○	◉	○	○	○	essential

Ryd markering

Does your paper address subitem 12a-i? *

Copy and paste relevant sections from the manuscript (include quotes in quotation marks "like this" to indicate direct quotes from your manuscript), or elaborate on this item by providing additional information not in the ms, or briefly explain why the item is not applicable/relevant for your study

Missing values were not imputed

12b) Methods for additional analyses, such as subgroup analyses and adjusted analyses

Does your paper address CONSORT subitem 12b? *

Copy and paste relevant sections from the manuscript (include quotes in quotation marks "like this" to indicate direct quotes from your manuscript), or elaborate on this item by providing additional information not in the ms, or briefly explain why the item is not applicable/relevant for your study

"Clinical outcomes were assessed in a post hoc analysis by linear mixed effect models [43]."

X26) REB/IRB Approval and Ethical Considerations [recommended as subheading under "Methods"] (not a CONSORT item)

X26-i) Comment on ethics committee approval

	1	2	3	4	5	
subitem not at all important	○	○	○	○	◉	essential

Ryd markering

Does your paper address subitem X26-i?

Copy and paste relevant sections from the manuscript (include quotes in quotation marks "like this" to indicate direct quotes from your manuscript), or elaborate on this item by providing additional information not in the ms, or briefly explain why the item is not applicable/relevant for your study

"The Ethical Committee of Denmark (H-16023499) and the Danish Data Protection Agency (I-Suite no.: 6262, NOH-2018-002) approved this study. Clinicaltrials.gov identifier: NCT03586622. All patients in the study gave written informed consent prior to their inclusion."

x26-ii) Outline informed consent procedures

Outline informed consent procedures e.g., if consent was obtained offline or online (how? Checkbox, etc.?), and what information was provided (see 4a-ii). See [6] for some items to be included in informed consent documents.

	1	2	3	4	5	
subitem not at all important	○	○	◉	○	○	essential

Ryd markering

Does your paper address subitem X26-ii?

Copy and paste relevant sections from the manuscript (include quotes in quotation marks "like this" to indicate direct quotes from your manuscript), or elaborate on this item by providing additional information not in the ms, or briefly explain why the item is not applicable/relevant for your study

Institutional affiliation was displayed to potential participants in information material (approved by the ethical committee of Denmark) and as well on the web-app IBS CC (https://ibs.constant-care.com/)

X26-iii) Safety and security procedures

Safety and security procedures, incl. privacy considerations, and any steps taken to reduce the likelihood or detection of harm (e.g., education and training, availability of a hotline)

	1	2	3	4	5	
subitem not at all important	○	◉	○	○	○	essential

Ryd markering

Does your paper address subitem X26-iii?

Copy and paste relevant sections from the manuscript (include quotes in quotation marks "like this" to indicate direct quotes from your manuscript), or elaborate on this item by providing additional information not in the ms, or briefly explain why the item is not applicable/relevant for your study

"Participants gained access to IBS CC via a log-in procedure that includes two-factor authentication. IBS CC (v 2.0) meets the requirements of the EU's General Data Protection Regulation (GDPR)......... Side effects, if any, were registered throughout the study period"

RESULTS

13a) For each group, the numbers of participants who were randomly assigned, received intended treatment, and were analysed for the primary outcome

NPT: The number of care providers or centers performing the intervention in each group and the number of patients treated by each care provider in each center

Does your paper address CONSORT subitem 13a? *

Copy and paste relevant sections from the manuscript (include quotes in quotation marks "like this" to indicate direct quotes from your manuscript), or elaborate on this item by providing additional information not in the ms, or briefly explain why the item is not applicable/relevant for your study

"Figure 2. Flow chart of inclusion, individualized treatment response and one-year follow-up"

13b) For each group, losses and exclusions after randomisation, together with reasons

Does your paper address CONSORT subitem 13b? (NOTE: Preferably, this is shown in a CONSORT flow diagram) *

Copy and paste relevant sections from the manuscript (include quotes in quotation marks "like this" to indicate direct quotes from your manuscript), or elaborate on this item by providing additional information not in the ms, or briefly explain why the item is not applicable/relevant for your study

"The number of IBS patients enrolled, randomizations to interventions, and responders and non-responders to interventions can be found in Figure 2. Twelve responded to LFD (57%) and eight (38%) completed the reintroduction to high-FODMAP foods. Thirteen (62%) responded to their first VSL#3® treatment; seven (33%) responded to multiple VSL#3® treatments. One-hundred-and-eighty fecal samples (156 from IBS and 24 from HC) were analyzed. ....Figure 2. Flow chart of inclusion, individualized treatment response and one-year follow-up"

13b-i) Attrition diagram

Strongly recommended: An attrition diagram (e.g., proportion of participants still logging in or using the intervention/comparator in each group plotted over time, similar to a survival curve) or other figures or tables demonstrating usage/dose/engagement.

	1	2	3	4	5	
subitem not at all important	○	○	◉	○	○	essential

Ryd markering

Does your paper address subitem 13b-i?

Copy and paste relevant sections from the manuscript or cite the figure number if applicable (include quotes in quotation marks "like this" to indicate direct quotes from your manuscript), or elaborate on this item by providing additional information not in the ms, or briefly explain why the item is not applicable/relevant for your study

"Figure 2. Flow chart of inclusion, individualized treatment response and one-year follow-up"

14a) Dates defining the periods of recruitment and follow-up

Does your paper address CONSORT subitem 14a? *

Copy and paste relevant sections from the manuscript (include quotes in quotation marks "like this" to indicate direct quotes from your manuscript), or elaborate on this item by providing additional information not in the ms, or briefly explain why the item is not applicable/relevant for your study

"Adult IBS patients (18 years or older) fulfilling the Rome III criteria and sub-diagnosed with either IBS-Diarrhea (IBS-D) or IBS-Mixed type (IBS-M) by a gastroenterologist were consecutively included at North Zealand University Hospital (August 23, 2018 to October 18, 2019).......This eHealth study was an open-label, randomized, crossover trial (for non-responders), with one year of follow-up (Figure 1)."

14a-i) Indicate if critical “secular events” fell into the study period

Indicate if critical “secular events” fell into the study period, e.g., significant changes in Internet resources available or “changes in computer hardware or Internet delivery resources”

	1	2	3	4	5	
subitem not at all important	◉	○	○	○	○	essential

Ryd markering

Does your paper address subitem 14a-i?

Copy and paste relevant sections from the manuscript (include quotes in quotation marks "like this" to indicate direct quotes from your manuscript), or elaborate on this item by providing additional information not in the ms, or briefly explain why the item is not applicable/relevant for your study

Not applicable for this study

14b) Why the trial ended or was stopped (early)

Does your paper address CONSORT subitem 14b? *

Copy and paste relevant sections from the manuscript (include quotes in quotation marks "like this" to indicate direct quotes from your manuscript), or elaborate on this item by providing additional information not in the ms, or briefly explain why the item is not applicable/relevant for your study

Not applicable for this study

15) A table showing baseline demographic and clinical characteristics for each group

NPT: When applicable, a description of care providers (case volume, qualification, expertise, etc.) and centers (volume) in each group

Does your paper address CONSORT subitem 15? *

Copy and paste relevant sections from the manuscript (include quotes in quotation marks "like this" to indicate direct quotes from your manuscript), or elaborate on this item by providing additional information not in the ms, or briefly explain why the item is not applicable/relevant for your study

"Table 1. Characteristics of participants at inclusion and upon randomization."

15-i) Report demographics associated with digital divide issues

In ehealth trials it is particularly important to report demographics associated with digital divide issues, such as age, education, gender, social-economic status, computer/Internet/ehealth literacy of the participants, if known.

	1	2	3	4	5	
subitem not at all important	○	○	◉	○	○	essential

Ryd markering

Does your paper address subitem 15-i? *

Copy and paste relevant sections from the manuscript (include quotes in quotation marks "like this" to indicate direct quotes from your manuscript), or elaborate on this item by providing additional information not in the ms, or briefly explain why the item is not applicable/relevant for your study

"Table 1. Characteristics of participants at inclusion and upon randomization."

16) For each group, number of participants (denominator) included in each analysis and whether the analysis was by original assigned groups

16-i) Report multiple “denominators” and provide definitions

Report multiple “denominators” and provide definitions: Report N’s (and effect sizes) “across a range of study participation [and use] thresholds” [1], e.g., N exposed, N consented, N used more than x times, N used more than y weeks, N participants “used” the intervention/comparator at specific pre-defined time points of interest (in absolute and relative numbers per group). Always clearly define “use” of the intervention.

	1	2	3	4	5	
subitem not at all important	○	○	◉	○	○	essential

Ryd markering

Does your paper address subitem 16-i? *

Copy and paste relevant sections from the manuscript (include quotes in quotation marks "like this" to indicate direct quotes from your manuscript), or elaborate on this item by providing additional information not in the ms, or briefly explain why the item is not applicable/relevant for your study

"The median time spent dieting by LFD responders (n=12) was 36 days (IQR: 28.00; 41.25) and for reintroduction (n=8) 296 days (IQR: 227.00; 305.75). For ‘true’ responders, i.e., participants who responded to multiple probiotic treatments (n=7) (see Figure 2), the median number of probiotic treatments needed for maintaining symptom control during the course of one year was three (IQR=2.25; 3.75) and the median (IQR) number of days between probiotic treatments was 46.5 (26.25; 65.75). Symptom remission, i.e., an IBS-SSS lower than 175, was achieved by eight (66.7%) LFD responders and five (71%) multiple probiotic responders, the latter corresponding to 12 out of 19 (63%) VSL#3® treatments bringing about symptom remission in probiotic responders.......As shown in Figure 3A, significant decreases in IBS-SSS were observed for LFD and probiotic responders relative to non-responders; both responder types were adherent to treatments (Figure 3C). However, no significant difference in effect size between the two treatments was found; the median IBS-SSS effect sizes were -126.50 (IQR: -196.75; -76.75) for LFD responders and -130.00 (IQR: -211.00; -70.50) for probiotic responders, P=1. The corresponding changes in QoL among LFD and probiotic responders was not significant compared to non-responders, nor were the median increases in QoL significant between the two responder groups, which were seven (IQR: 2.75; 14.20) for LFD responders and three (IQR: 0-16.00) for probiotic responders, P=1."

16-ii) Primary analysis should be intent-to-treat

Primary analysis should be intent-to-treat, secondary analyses could include comparing only “users”, with the appropriate caveats that this is no longer a randomized sample (see 18-i).

	1	2	3	4	5	
subitem not at all important	○	○	○	◉	○	essential

Ryd markering

Does your paper address subitem 16-ii?

Copy and paste relevant sections from the manuscript (include quotes in quotation marks "like this" to indicate direct quotes from your manuscript), or elaborate on this item by providing additional information not in the ms, or briefly explain why the item is not applicable/relevant for your study

The primary analysis for Low FODMAP responders and reintroduction were reported as ITT

17a) For each primary and secondary outcome, results for each group, and the estimated effect size and its precision (such as 95% confidence interval)

Does your paper address CONSORT subitem 17a? *

Copy and paste relevant sections from the manuscript (include quotes in quotation marks "like this" to indicate direct quotes from your manuscript), or elaborate on this item by providing additional information not in the ms, or briefly explain why the item is not applicable/relevant for your study

Shown i figures 3 and 4 "As shown in Figure 3A, significant decreases in IBS-SSS were observed for LFD and probiotic responders relative to non-responders; both responder types were adherent to treatments (Figure 3C). However, no significant difference in effect size between the two treatments was found; the median IBS-SSS effect sizes were -126.50 (IQR: -196.75; -76.75) for LFD responders and -130.00 (IQR: -211.00; -70.50) for probiotic responders, P=1"

17a-i) Presentation of process outcomes such as metrics of use and intensity of use

In addition to primary/secondary (clinical) outcomes, the presentation of process outcomes such as metrics of use and intensity of use (dose, exposure) and their operational definitions is critical. This does not only refer to metrics of attrition (13-b) (often a binary variable), but also to more continuous exposure metrics such as “average session length”. These must be accompanied by a technical description how a metric like a “session” is defined (e.g., timeout after idle time) [1] (report under item 6a).

	1	2	3	4	5	
subitem not at all important	(●)	( )	( )	( )	( )	essential

Ryd markering

Does your paper address subitem 17a-i?

Copy and paste relevant sections from the manuscript (include quotes in quotation marks "like this" to indicate direct quotes from your manuscript), or elaborate on this item by providing additional information not in the ms, or briefly explain why the item is not applicable/relevant for your study

Entries were not counted, however, web-consultations and text messages via IBS CC were counted. "During the one-year study period, originating from both eCare provider and participants (n=33, including HCs), 887 web consultations and 781 text messages were registered."

17b) For binary outcomes, presentation of both absolute and relative effect sizes is recommended

Does your paper address CONSORT subitem 17b? *

Copy and paste relevant sections from the manuscript (include quotes in quotation marks "like this" to indicate direct quotes from your manuscript), or elaborate on this item by providing additional information not in the ms, or briefly explain why the item is not applicable/relevant for your study

Only adherence was considered as a binary outcome measure in this study. "During reintroduction to high-FODMAP foods, patients deviated significantly from the principles of the LFD (Figure 3C, P=.004), resulting in a small but significant median increase in symptom severity relative to the time period where they were on the LFD (Figure 3A)."

18) Results of any other analyses performed, including subgroup analyses and adjusted analyses, distinguishing pre-specified from exploratory

Does your paper address CONSORT subitem 18? *

Copy and paste relevant sections from the manuscript (include quotes in quotation marks "like this" to indicate direct quotes from your manuscript), or elaborate on this item by providing additional information not in the ms, or briefly explain why the item is not applicable/relevant for your study

"These results are generally substantiated by fitting linear mixed effect models of clinical metadata from the IBS CC database (i.e., not considering delta values alone) to estimate effect sizes, e.g., for responder groups. These post hoc models of QoL showed that LFD responders saw significant increases in their QoL while on the LFD (by an estimated 9.08), and even more during reintroduction (13.48) relative to their baseline QoL (an estimated 60.20). The model estimating the effect on severity scoring showed significant decreases for all responder types, including probiotic responders between active treatments. Outputs from the linear mixed effect models are shown in Multimedia Appendix 2."

18-i) Subgroup analysis of comparing only users

A subgroup analysis of comparing only users is not uncommon in ehealth trials, but if done, it must be stressed that this is a self-selected sample and no longer an unbiased sample from a randomized trial (see 16-iii).

	1	2	3	4	5	
subitem not at all important	◉	○	○	○	○	essential

Ryd markering

Does your paper address subitem 18-i?

Copy and paste relevant sections from the manuscript (include quotes in quotation marks "like this" to indicate direct quotes from your manuscript), or elaborate on this item by providing additional information not in the ms, or briefly explain why the item is not applicable/relevant for your study

Sub-group analyses were not performed in relation to the eHealth intervention. Sub-group analyses have been performed e.g. based on diagnoses and responder types in relation to the microbiota data and results shown in multimedia Appendix 2.

19) All important harms or unintended effects in each group

(for specific guidance see CONSORT for harms)

Does your paper address CONSORT subitem 19? *

Copy and paste relevant sections from the manuscript (include quotes in quotation marks "like this" to indicate direct quotes from your manuscript), or elaborate on this item by providing additional information not in the ms, or briefly explain why the item is not applicable/relevant for your study

"No adverse events were registered for either intervention"

19-i) Include privacy breaches, technical problems

Include privacy breaches, technical problems. This does not only include physical “harm” to participants, but also incidents such as perceived or real privacy breaches [1], technical problems, and other unexpected/unintended incidents. “Unintended effects” also includes unintended positive effects [2].

	1	2	3	4	5	
subitem not at all important	○	○	◉	○	○	essential

Ryd markering

Does your paper address subitem 19-i?

Copy and paste relevant sections from the manuscript (include quotes in quotation marks "like this" to indicate direct quotes from your manuscript), or elaborate on this item by providing additional information not in the ms, or briefly explain why the item is not applicable/relevant for your study

This has been evaluated by the participant and described in Multimedia Appendix 2.

19-ii) Include qualitative feedback from participants or observations from staff/researchers

Include qualitative feedback from participants or observations from staff/researchers, if available, on strengths and shortcomings of the application, especially if they point to unintended/unexpected effects or uses. This includes (if available) reasons for why people did or did not use the application as intended by the developers.

	1	2	3	4	5	
subitem not at all important	○	○	○	●	○	essential

Ryd markering

Does your paper address subitem 19-ii?

Copy and paste relevant sections from the manuscript (include quotes in quotation marks "like this" to indicate direct quotes from your manuscript), or elaborate on this item by providing additional information not in the ms, or briefly explain why the item is not applicable/relevant for your study

"The evaluation at one-year follow-up showed overall satisfaction with the study, the support received during the study, and with IBS CC itself (further details can be found in Multimedia Appendix 2)"

DISCUSSION

22) Interpretation consistent with results, balancing benefits and harms, and considering other relevant evidence

NPT: In addition, take into account the choice of the comparator, lack of or partial blinding, and unequal expertise of care providers or centers in each group

22-i) Restate study questions and summarize the answers suggested by the data, starting with primary outcomes and process outcomes (use)

Restate study questions and summarize the answers suggested by the data, starting with primary outcomes and process outcomes (use).

	1	2	3	4	5	
subitem not at all important	○	○	○	○	◉	essential

Ryd markering

Does your paper address subitem 22-i? *

Copy and paste relevant sections from the manuscript (include quotes in quotation marks "like this" to indicate direct quotes from your manuscript), or elaborate on this item by providing additional information not in the ms, or briefly explain why the item is not applicable/relevant for your study

"In this study, we have shown that the new and expanded IBS Constant Care web application for long-term management of IBS is practical, safe and useful in clinical decision-making. This was demonstrated by two year-long treatment interventions, the low FODMAP diet (with subsequent reintroduction of high-FODMAP foods for LFD responders) and a probiotic treatment (repeated for four weeks every time a symptom flare-up occurred in probiotic responders). These therapeutics were found to be equally good at managing IBS symptoms in the short- and long-term. We also investigated participants' fecal microbiota, in order to try to predict response to the interventions (in the hope of being able to individualize future treatments for IBS); however, we were unable to make these predictions. "

22-ii) Highlight unanswered new questions, suggest future research

Highlight unanswered new questions, suggest future research.

	1	2	3	4	5	
subitem not at all important	○	○	○	◉	○	essential

Ryd markering

Does your paper address subitem 22-ii?

Copy and paste relevant sections from the manuscript (include quotes in quotation marks "like this" to indicate direct quotes from your manuscript), or elaborate on this item by providing additional information not in the ms, or briefly explain why the item is not applicable/relevant for your study

"Due to the small sample size of the study, larger ones are needed to confirm our results and would, ideally, include other medical options for managing IBS, especially for non-responders to the LFD and probiotic treatments."

20) Trial limitations, addressing sources of potential bias, imprecision, and, if relevant, multiplicity of analyses

20-i) Typical limitations in ehealth trials

Typical limitations in ehealth trials: Participants in ehealth trials are rarely blinded. Ehealth trials often look at a multiplicity of outcomes, increasing risk for a Type I error. Discuss biases due to non-use of the intervention/usability issues, biases through informed consent procedures, unexpected events.

	1	2	3	4	5	
subitem not at all important	○	○	○	●	○	essential

Ryd markering

Does your paper address subitem 20-i? *

Copy and paste relevant sections from the manuscript (include quotes in quotation marks "like this" to indicate direct quotes from your manuscript), or elaborate on this item by providing additional information not in the ms, or briefly explain why the item is not applicable/relevant for your study

"The strength of this study is its use of an updated and expanded eHealth web application to monitor and treat IBS patients according to their treatment response across one year of follow-up. To our knowledge, this is the first eHealth trial to monitor the effect prospectively and longitudinally of multiple probiotic treatments and LFD with subsequent re-introduction of foods high in FODMAPs, and to compare responses to patients' microbiota.
A limitation of the study is its sample size, and that it is based on a single study center."

21) Generalisability (external validity, applicability) of the trial findings

NPT: External validity of the trial findings according to the intervention, comparators, patients, and care providers or centers involved in the trial

21-i) Generalizability to other populations

Generalizability to other populations: In particular, discuss generalizability to a general Internet population, outside of a RCT setting, and general patient population, including applicability of the study results for other organizations

	1	2	3	4	5	
subitem not at all important	○	○	○	●	○	essential

Ryd markering

Does your paper address subitem 21-i?

Copy and paste relevant sections from the manuscript (include quotes in quotation marks "like this" to indicate direct quotes from your manuscript), or elaborate on this item by providing additional information not in the ms, or briefly explain why the item is not applicable/relevant for your study

"Other researchers have suggested that web-based/app-based LFD and probiotic treatments may provide therapeutic benefits [62] and should be more widely implemented for IBS management [13]. This study supports these existing results and recommendations and adds further evidence of using eHealth for clinical decision-making and long-term management."

21-ii) Discuss if there were elements in the RCT that would be different in a routine application setting

Discuss if there were elements in the RCT that would be different in a routine application setting (e.g., prompts/reminders, more human involvement, training sessions or other co-interventions) and what impact the omission of these elements could have on use, adoption, or outcomes if the intervention is applied outside of a RCT setting.

	1	2	3	4	5	
subitem not at all important	●	○	○	○	○	essential

Ryd markering

Does your paper address subitem 21-ii?

Copy and paste relevant sections from the manuscript (include quotes in quotation marks "like this" to indicate direct quotes from your manuscript), or elaborate on this item by providing additional information not in the ms, or briefly explain why the item is not applicable/relevant for your study

This has been addressed in Multimedia Appendix 2. "The evaluation at one-year follow-up showed overall satisfaction with the study, the support received during the study, and with IBS CC itself (further details can be found in Multimedia Appendix 2)".

OTHER INFORMATION

23) Registration number and name of trial registry

Does your paper address CONSORT subitem 23? *

Copy and paste relevant sections from the manuscript (include quotes in quotation marks "like this" to indicate direct quotes from your manuscript), or elaborate on this item by providing additional information not in the ms, or briefly explain why the item is not applicable/relevant for your study

"The Ethical Committee of Denmark (H-16023499) and the Danish Data Protection Agency (I-Suite no.: 6262, NOH-2018-002) approved this study. Clinicaltrials.gov identifier: NCT03586622. All patients in the study gave written informed consent prior to their inclusion."

24) Where the full trial protocol can be accessed, if available

Does your paper address CONSORT subitem 24? *

Cite a Multimedia Appendix, other reference, or copy and paste relevant sections from the manuscript (include quotes in quotation marks "like this" to indicate direct quotes from your manuscript), or elaborate on this item by providing additional information not in the ms, or briefly explain why the item is not applicable/relevant for your study

Via the Ethical Committee of Denmark

25) Sources of funding and other support (such as supply of drugs), role of funders

Does your paper address CONSORT subitem 25? *

Copy and paste relevant sections from the manuscript (include quotes in quotation marks "like this" to indicate direct quotes from your manuscript), or elaborate on this item by providing additional information not in the ms, or briefly explain why the item is not applicable/relevant for your study

"Grants supporting this study were provided by North Zealand University Hospital, Ferring Pharmaceuti-cals, Handelsgartner Ove William Buhl Olesen and ægtefælle fru Edith Buhl Olesens memorial fund, Dagmar and Ivar Enevoldsen memorial fund and the Crohn's & Colitis Patient Society, Denmark. This study was also supported in part by Unseen Bio Aps and Calpro AS. "

X27) Conflicts of Interest (not a CONSORT item)

X27-i) State the relation of the study team towards the system being evaluated

In addition to the usual declaration of interests (financial or otherwise), also state the relation of the study team towards the system being evaluated, i.e., state if the authors/evaluators are distinct from or identical with the developers/sponsors of the intervention.

	1	2	3	4	5	
subitem not at all important	○	○	○	●	○	essential

Ryd markering

Does your paper address subitem X27-i?

Copy and paste relevant sections from the manuscript (include quotes in quotation marks "like this" to indicate direct quotes from your manuscript), or elaborate on this item by providing additional information not in the ms, or briefly explain why the item is not applicable/relevant for your study

"DVA has received grants from the Crohn’s & Colitis Patient Society, Denmark, North Zealand University Hospital, Ferring Pharmaceuti¬cals, Handelsgartner Ove William Buhl Olesen and ægtefælle fru Edith Buhl Olsen’s memorial fund, Dagmar and Ivar Enevoldsen memorial fund and non-financial support from Calpro AS. PW has received consulting fees from Vifor Pharma Nordiska AB, grants from Ferring lægemidler and Tillotts Pharma AG, as well as non-financial support from Janssen-Cilag A/S, Calpro AS, Pharmacosmos A/S and Vifor Pharma Nordiska AB. MB has received other financial support from AbbVie, Tillotts, Takeda, MSD and Pfizer. KP is an employee of Ferring Pharmaceuti¬cals. MEB and CL are owners of Unseen Bio Aps. JB has received personal fees from AbbVie, Janssen-Cilag, Celgene, Samsung Biopies, MSD, Pfizer, and Coloplast, as well as grants and personal fees from Takeda and Tillots Pharma. PM has received financial, as well as non-financial, support from AbbVie, AstraZeneca, Pfizer, Janssen-Cilag, Takeda, Tillotts Pharma, MSD, Ferring Pharmaceuti¬cals and Calpro AS. The remaining authors have no interests to declare"

About the CONSORT EHEALTH checklist

As a result of using this checklist, did you make changes in your manuscript? *

- ○ yes, major changes
- ◉ yes, minor changes
- ○ no

What were the most important changes you made as a result of using this checklist?

E.g. I had forgotten to include the export function and the IBS CC database in our manuscript. This has now been included

How much time did you spend on going through the checklist INCLUDING making changes in your manuscript *

I have used one day to fill in this form

As a result of using this checklist, do you think your manuscript has improved? *

- (x) yes
- ( ) no
- ( ) Andet:

Would you like to become involved in the CONSORT EHEALTH group?

This would involve for example becoming involved in participating in a workshop and writing an "Explanation and Elaboration" document

- (x) yes
- ( ) no
- ( ) Andet:

Ryd markering

Any other comments or questions on CONSORT EHEALTH

Dit svar

STOP - Save this form as PDF before you click submit

To generate a record that you filled in this form, we recommend to generate a PDF of this page (on a Mac, simply select "print" and then select "print as PDF") before you submit it.

When you submit your (revised) paper to JMIR, please upload the PDF as supplementary file.

Don't worry if some text in the textboxes is cut off, as we still have the complete information in our database. Thank you!

Final step: Click submit !

Click submit so we have your answers in our database!

Send

Indsend aldrig adgangskoder via Google Analyse.

Dette indhold er hverken oprettet eller godkendt af Google. Rapportér misbrug - Servicevilkår - Privatlivspolitik

Google Analyse
