# Supplementary material for: Long-Term Effects of a Web-Based Low-FODMAP Diet Versus Probiotic Treatment for Irritable Bowel Syndrome, Including Shotgun Analyses of Microbiota: Randomized, Double-Crossover Clinical Trial
Source: J Med Internet Res. 2021 Dec 14;23(12):e30291. doi: 10.2196/30291 (PMC8715363; doi:10.2196/30291)
Supplement: Multimedia Appendix 2 [file jmir_v23i12e30291_app2.pdf]

Multimedia Appendix 2. Supplementary elaborations, tables, and figures

Contents

Elaborations 2

Elaboration 1 on reintroduction of foods high in FODMAPs 2

Elaboration 2 on R Packages used in this study 3

References 3

Elaboration 3 on LME (linear mixed effect models) 6

Output, IBS-SSS 6

Output, IBS-QoL 8

Output, Compliance 10

Output, Bowel frequency 11

Tables 13

Table 1. Instructions to patients of how often they should monitor themselves at home using the web application, IBS Constant Care 13

Figures 14

Figure 1. One-year disease course of a Low FODMAP diet responder 14

Figure 2. One-year disease course of a probiotic responder 15

Figure 3. Change in fecal calprotectin and disease course types based on responder types 16

Figure 4. Patient reported evaluation at one-year follow up 17

Figure 5. Alpha diversities for healthy controls and IBS patients 18

Figure 6. Inter- and intra-individual Bray Curtis dissimilarity 19

Figure 7. Intra-individual Bray-Curtis dissimilarity and UMAP plot according to diagnoses 20

Figure 8. Intra-individual Bray-Curtis dissimilarity and UMAP plot according to responder types 21

Figure 9. Alpha diversities for responder types including the corresponding output of LME 22

Elaborations

Elaboration 1 on reintroduction of foods high in FODMAPs

Responders to the LFD were taught how to reintroduce foods high in FODMAPs using the food preference approach, while considering their usual/preferred dietary intake. For instance, a tall male basketball player would like to know if 6 pieces (preferred daily intake) of rye bread could be tolerated a day – in this case he would be instructed to reintroduce 2 pieces the first day (and mark this as green in the reintroduction module in the IBS CC before proceeding), 4 the next day, and 6 pieces on the third day. If symptoms occurred on day 2, they were instructed to mark this quantity in the reintroduction module in IBS CC with either a yellow colour and only reintroduce 5 pieces on the third day, or mark it red and stop the reintroduction process at day 2. Patients were advised to stick to LFD principles while reintroducing high-FODMAP food. Patients were allowed, if they had time remaining before the one-year follow-up, to reintroduce combined foods or meals, e.g., ryebread and hummus. Patients were also allowed to re-test foods that they had previously failed to tolerate during the reintroduction.

Patients returned home from the reintroduction consultation (approx. one-hour) with a personalized, prioritised list of foods to reintroduce, including their quantities. They were advised to start reintroducing a new food on the list every Monday and were further instructed in using the reintroduction module in IBS CC (see figure below), where they could enter the name and quantity of the food reintroduced for all 3 days, and label the foods individually as either green, yellow or red (depending on their tolerance). In the end, patients could export (into Excel) the list of their reintroduced foods according to color.

A shortened example of the reintroduction module in IBS CC is shown below. Patients could sort data according to date (=Dato) or results (=Resultat). In the figure below, foods are sorted according to results (green, yellow and red). Fødevarer=foods, Enhed=unit, 1. dag-3.dag=first-to-third day. Patients made an overall evaluation for every food reintroduced in the Resultat column and marked them as either green=tolerated, yellow=tolerated to some extent, red=not tolerated.

Fødevarer jeg vil forsøge at gen-introducere (sortér efter kolonne med blå kolonnenavne ved at klikke på kolonnenavnet):

Fødevare	Dato	Enhed	1. dag	2. dag	3. dag	Resultat
Apple juice	06-01-2019	dl	Mængde: 1 dl<br>Tåles	Mængde: 2 dl<br>Tåles	Mængde: 3 dl<br>Tåles	Tåles
Asparagus	20-01-2019	stk.	Mængde: 1 stk.<br>Tåles	Mængde: 2 stk.<br>Tåles	Mængde: 3 stk.<br>Tåles	Tåles
Broccoli	03-02-2019	g	Mængde: 50 g<br>Tåles	Mængde: 100 g<br>Tåles i nogen grad	Mængde: 150 g<br>Tåles i nogen grad	Tåles i nogen grad
Leek	04-03-2019	stk.	Mængde: 1 stk.<br>Tåles	Mængde: 1,5 stk.<br>Tåles i nogen grad	Mængde: 2 stk.<br>Tåles i nogen grad	Tåles i nogen grad
Bread	18-02-2019	stk.	Mængde: 0,5 stk.<br>Tåles	Mængde: 1 stk.<br>Tåles ikke	Mængde: 1 stk.<br>Tåles ikke	Tåles ikke
Mushrooms	18-03-2019	g	Mængde: 40 g<br>Tåles ikke	Mængde:<br>Tåles ikke	Mængde:<br>Tåles ikke	Tåles ikke

Elaboration 2 on R packages used in this study

- base (R Core Team 2021a)
- car (Fox and Weisberg 2019)
- colorspace (Stauffer et al. 2009)
- cowplot (Wilke 2020)
- dabestr (Ho et al. 2019)
- dplyr (Wickham et al. 2021)
- ggfittext (Wilkins 2021)
- ggforce (Pedersen 2021)
- ggplot2 (Wickham 2016)
- ggpubr (Kassambara 2020)
- ggsignif (Ahlmann-Eltze and Patil 2021)
- ggstatsplot (Patil 2018)
- knitr (Xie 2014)
- lmerTest (Kuznetsova, Brockhoff, and Christensen 2017)
- magrittr (Bache and Wickham 2020)
- pairwiseComparisons (Patil 2019)
- phyloseq (McMurdie and Holmes 2013)
- plotly (Sievert 2020)
- purrr (Henry and Wickham 2020)
- rcartocolor (Nowosad 2018)
- readr (Wickham and Hester 2020)
- report (Makowski et al. 2020)
- Rtsne (Krijthe 2015)
- stats (R Core Team 2021b)
- testthat (Wickham 2011)
- tibble (Müller and Wickham 2021)
- tidyr (Wickham 2021)
- uwot (Melville 2020)
- vegan (Oksanen et al. 2020)
- writexl (Ooms 2020)
- assertthat (Wickham 2019a)
- grateful (Rodríguez-Sánchez and Hutchins 2020)
- lubridate (Grolemund and Wickham 2011)
- mustashe (Cook 2020)
- stringr (Wickham 2019b)
- corncob (Martin, Witten, and Willis 2021)

References

Ahlmann-Eltze, Constantin, and Indrajeet Patil. 2021. *Ggsignif: Significance Brackets for 'Ggplot2'*. https://CRAN.R-project.org/package=ggsignif.

Bache, Stefan Milton, and Hadley Wickham. 2020. *Magrittr: A Forward-Pipe Operator for r*. https://CRAN.R-project.org/package=magrittr.

Cook, Joshua H. 2020. *Mustashe: Stash and Load Objects*. https://CRAN.R-project.org/package=mustashe.

Fox, John, and Sanford Weisberg. 2019. *An R Companion to Applied Regression*. Third. Thousand Oaks CA: Sage. https://socialsciences.mcmaster.ca/jfox/Books/Companion/.

Grolemund, Garrett, and Hadley Wickham. 2011. “Dates and Times Made Easy with lubridate.” *Journal of Statistical Software* 40 (3): 1–25. https://www.jstatsoft.org/v40/i03/.

Henry, Lionel, and Hadley Wickham. 2020. *Purrr: Functional Programming Tools*. https://CRAN.R-project.org/package=purrr.

Ho, Joses, Tayfun Tumkaya, Sameer Aryal, Hyungwon Choi, and Adam Claridge-Chang. 2019. “Moving Beyond p Values: Everyday Data Analysis with Estimation Plots.” https://doi.org/10.1038/s41592-019-0470-3.

Kassambara, Alboukadel. 2020. *Ggpubr: ’Ggplot2’ Based Publication Ready Plots*. https://CRAN.R-project.org/package=ggpubr.

Krijthe, Jesse H. 2015. *Rtsne: T-Distributed Stochastic Neighbor Embedding Using Barnes-Hut Implementation*. https://github.com/jkrijthe/Rtsne.

Kuznetsova, Alexandra, Per B. Brockhoff, and Rune H. B. Christensen. 2017. “lmerTest Package: Tests in Linear Mixed Effects Models.” *Journal of Statistical Software* 82 (13): 1–26. https://doi.org/10.18637/jss.v082.i13.

Makowski, Dominique, Lüdecke, Daniel, Ben-Shachar, and Mattan S. 2020. “Automated Reporting as a Practical Tool to Improve Reproducibility and Methodological Best Practices Adoption.” *CRAN*. https://github.com/easystats/report.

Martin, Bryan D, Daniela Witten, and Amy D Willis. 2021. *Corncob: Count Regression for Correlated Observations with the Beta-Binomial*. https://CRAN.R-project.org/package=corncob.

McMurdie, Paul J., and Susan Holmes. 2013. “Phyloseq: An r Package for Reproducible Interactive Analysis and Graphics of Microbiome Census Data.” *PLoS ONE* 8 (4): e61217. http://dx.plos.org/10.1371/journal.pone.0061217.

Melville, James. 2020. *Uwot: The Uniform Manifold Approximation and Projection (UMAP) Method for Dimensionality Reduction*. https://CRAN.R-project.org/package=uwot.

Müller, Kirill, and Hadley Wickham. 2021. *Tibble: Simple Data Frames*. https://CRAN.R-project.org/package=tibble.

Nowosad, Jakub. 2018. *’CARTOColors’ Palettes*. https://nowosad.github.io/rcartocolor.

Oksanen, Jari, F. Guillaume Blanchet, Michael Friendly, Roeland Kindt, Pierre Legendre, Dan McGlinn, Peter R. Minchin, et al. 2020. *Vegan: Community Ecology Package*. https://CRAN.R-project.org/package=vegan.

Ooms, Jeroen. 2020. *Writexl: Export Data Frames to Excel ’Xlsx’ Format*. https://CRAN.R-project.org/package=writexl.

Patil, Indrajeet. 2018. “ggstatsplot: ’Ggplot2’ Based Plots with Statistical Details.” *CRAN*. https://doi.org/10.5281/zenodo.2074621.

———. 2019. *pairwiseComparisons: Multiple Pairwise Comparison Tests*. https://CRAN.R-project.org/package=pairwiseComparisons.

Pedersen, Thomas Lin. 2021. *Ggforce: Accelerating ’Ggplot2’*. https://CRAN.R-project.org/package=ggforce.

R Core Team. 2021a. *R: A Language and Environment for Statistical Computing*. Vienna, Austria: R Foundation for Statistical Computing. https://www.R-project.org/.

———. 2021b. *R: A Language and Environment for Statistical Computing*. Vienna, Austria: R Foundation for Statistical Computing. https://www.R-project.org/.

Rodríguez-Sánchez, Francisco, and Shaurita D. Hutchins. 2020. *Grateful: Facilitate Citation of r Packages*. https://github.com/Pakillo/grateful.

Sievert, Carson. 2020. *Interactive Web-Based Data Visualization with r, Plotly, and Shiny*. Chapman; Hall/CRC. https://plotly-r.com.

Stauffer, Reto, Georg J. Mayr, Markus Dabernig, and Achim Zeileis. 2009. "Somewhere over the Rainbow: How to Make Effective Use of Colors in Meteorological Visualizations." *Bulletin of the American Meteorological Society* 96 (2): 203–16. https://doi.org/10.1175/BAMS-D-13-00155.1.

Wickham, Hadley. 2011. "Testthat: Get Started with Testing." *The R Journal* 3: 5–10. https://journal.r-project.org/archive/2011-1/RJournal_2011-1_Wickham.pdf.

———. 2016. *Ggplot2: Elegant Graphics for Data Analysis*. Springer-Verlag New York. https://ggplot2.tidyverse.org.

———. 2019a. *Assertthat: Easy Pre and Post Assertions*. https://CRAN.R-project.org/package=assertthat.

———. 2019b. *Stringr: Simple, Consistent Wrappers for Common String Operations*. https://CRAN.R-project.org/package=stringr.

———. 2021. *Tidyr: Tidy Messy Data*. https://CRAN.R-project.org/package=tidyr.

Wickham, Hadley, Romain François, Lionel Henry, and Kirill Müller. 2021. *Dplyr: A Grammar of Data Manipulation*. https://CRAN.R-project.org/package=dplyr.

Wickham, Hadley, and Jim Hester. 2020. *Readr: Read Rectangular Text Data*. https://CRAN.R-project.org/package=readr.

Wilke, Claus O. 2020. *Cowplot: Streamlined Plot Theme and Plot Annotations for 'Ggplot2'*. https://CRAN.R-project.org/package=cowplot.

Wilkins, David. 2021. *Ggfittext: Fit Text Inside a Box in 'Ggplot2'*. https://CRAN.R-project.org/package=ggfittext.

Xie, Yihui. 2014. "Knitr: A Comprehensive Tool for Reproducible Research in R." In *Implementing Reproducible Computational Research*, edited by Victoria Stodden, Friedrich Leisch, and Roger D. Peng. Chapman; Hall/CRC. http://www.crcpress.com/product/isbn/9781466561595.

Elaboration 3 on LME (linear mixed effect models)

Outputs (IBS-SSS, IBS-QoL, compliance and bowel frequency) of fitting linear mixed effects models (using restricted maximum likelihood (REML)) for Clinical Constant Care metadata are shown below.

Abbreviations used in the LME: Low FODMAP responder= LFD R, Reintroduction=Reintro, Probiotic responder=Prob R, In between active probiotic treatments= Prob CC, Non-responder=NR, symptom severity score= IBS-SSS or SSS, quality of life= IBS-QoL or QoL, Compliance to interventions= FARS/MARS, Bristol stool chart=Bristol.

Output, IBS-SSS

We fitted a linear mixed model (estimated using REML and nloptwrap optimizer) to predict sss with group, frequency, bristol, age and answer_day (formula: sss ~ group + frequency + bristol + age + group:answer_day). The model included random_id as random effect (formula: ~1 | random_id). The model's total explanatory power is substantial (conditional R2 = 0.77) and the part related to the fixed effects alone (marginal R2) is of 0.42. The model's intercept, corresponding to group = Baseline, frequency = 0, bristol = 1, age = [1,40) and answer_day = 0, is at 285.79 (95% CI [231.97, 339.62], t(494) = 10.41, *P* < .001). Within this model

Parameter	Coefficient	CI	CI_low	CI_high	t	df_error	*P*	Std_Coefficient	Fit
(Intercept)	285.7948	0.95	231.966	339.624	10.4061	494	0.00e+00	1.0133	
group [LFD R]	-62.0359	0.95	-95.977	-28.095	-3.5824	494	3.40e-04	-1.1264	
group [Re-Intro]	-110.8986	0.95	-136.203	-85.594	-8.5897	494	0.00e+00	-0.6349	
group [Prob R]	-45.7004	0.95	-74.733	-16.668	-3.0852	494	2.03e-03	-1.2638	
group [Prob CC]	-95.7971	0.95	-122.474	-69.120	-7.0382	494	0.00e+00	-0.5546	
group [NR]	-0.7346	0.95	-48.248	46.778	-0.0303	494	9.76e-01	0.4458	
frequency	20.8040	0.95	15.777	25.831	8.1119	494	0.00e+00	0.2844	
bristol [2]	-7.2139	0.95	-44.620	30.192	-0.3780	494	7.05e-01	-0.0644	
bristol [3]	-17.7909	0.95	-51.834	16.252	-1.0243	494	3.06e-01	-0.1588	
bristol [4]	-41.1479	0.95	-71.982	-10.313	-2.6155	494	8.91e-03	-0.3673	
bristol [5]	-13.6682	0.95	-44.890	17.553	-0.8580	494	3.91e-01	-0.1220	
bristol [6]	10.6945	0.95	-20.888	42.277	0.6637	494	5.07e-01	0.0955	
bristol [7]	7.2339	0.95	-34.757	49.225	0.3377	494	7.36e-01	0.0646	

age [40-Inf]	-100.4923	0.95	-161.992	-38.992	-3.2026	494	1.36e-03	-0.8970	
group [Baseline] * answer_day	-0.8511	0.95	-1.214	-0.488	-4.5995	494	4.20e-06	-0.5021	
group [LFD R] * answer_day	-2.1143	0.95	-3.372	-0.857	-3.2962	494	9.80e-04	-1.2473	
group [Re-Intro] * answer_day	-0.0679	0.95	-0.183	0.047	-1.1582	494	2.47e-01	-0.0400	
group [Prob R] * answer_day	-2.7389	0.95	-3.778	-1.699	-5.1642	494	2.00e-07	-1.6157	
group [Prob CC] * answer_day	-0.1883	0.95	-0.423	0.046	-1.5754	494	1.15e-01	-0.1111	
group [NR] * answer_day	0.1468	0.95	-1.633	1.927	0.1616	494	8.72e-01	0.0866	
R2 (conditional)									0.773
R2 (marginal)									0.416
Sigma									58.221

Take-home message: This model shows that LFD responders, on average, experienced decreases in their severity score by an estimated 62 points compared to their intercept score (average: 285). During reintroduction, the estimated decrease was 110 and also highly significant. For probiotic responders, the estimated decrease was 45, and in between active treatments it was 95, compared to their baseline, both of which are statistically significant.

Output, IBS-QoL

We fitted a linear mixed model (estimated using REML and nloptwrap optimizer) to predict qol with group, frequency, bristol, age, smoking and answer_day (formula: qol ~ group + frequency + bristol + age + smoking + answer_day:group). The model included random_id as random effect (formula: ~1 | random_id). The model's total explanatory power is substantial (conditional R2 = 0.91) and the part related to the fixed effects alone (marginal R2) is of 0.51. The model's intercept, corresponding to group = Baseline, frequency = 0, bristol = 1, age = [1,40), smoking = never and answer_day = 0, is at 60.20 (95% CI [47.89, 72.51], t(218) = 9.59, *P* < .001). Within this model

Parameter	Coefficient	CI	CI_low	CI_high	t	df_error	*P*	Std_Coefficient	Fit
(Intercept)	6.02e+01	0.95	47.89426	72.5121	9.586	218	0.00e+00	-0.50484	
group [LFD R]	9.08e+00	0.95	3.15265	14.9997	3.003	218	2.67e-03	0.15673	
group [Re-Intro]	1.35e+01	0.95	8.14296	18.8197	4.950	218	7.00e-07	0.38359	
group [Prob R]	2.80e+00	0.95	-2.25374	7.8628	1.087	218	2.77e-01	0.25646	
group [Prob CC]	6.97e+00	0.95	2.33892	11.5981	2.950	218	3.18e-03	0.04862	
group [NR]	1.97e+00	0.95	-6.90012	10.8347	0.435	218	6.64e-01	-0.22643	
frequency	-3.59e+00	0.95	-4.71619	-2.4547	-6.215	218	0.00e+00	-0.21661	
bristol [2]	-6.22e-01	0.95	-8.17740	6.9325	-0.161	218	8.72e-01	-0.02486	
bristol [3]	9.68e-01	0.95	-6.28753	8.2236	0.262	218	7.94e-01	0.03866	
bristol [4]	-3.70e-01	0.95	-6.91084	6.1715	-0.111	218	9.12e-01	-0.01476	
bristol [5]	-2.01e+00	0.95	-8.79966	4.7863	-0.579	218	5.63e-01	-0.08015	
bristol [6]	-3.43e+00	0.95	-10.19839	3.3362	-0.994	218	3.20e-01	-0.13704	
bristol [7]	-3.88e+00	0.95	-13.22122	5.4583	-0.815	218	4.15e-01	-0.15503	
age [40-Inf]	3.03e+01	0.95	16.50650	44.0485	4.309	218	1.64e-05	1.20931	
smoking [currently]	-2.46e+01	0.95	-48.75283	-0.4125	-1.993	218	4.62e-02	-0.98185	
smoking [previously]	-1.29e+01	0.95	-28.71669	2.8935	-1.601	218	1.09e-01	-0.51570	
group [Baseline] * answer_day	1.09e-01	0.95	0.03138	0.1869	2.751	218	5.95e-03	0.33490	

group [LFD R] * answer_day	1.05e-02	0.95	-0.18633	0.2074	0.105	218	9.16e-01	0.03233	
group [Re-Intro] * answer_day	3.49e-02	0.95	0.01464	0.0552	3.374	218	7.41e-04	0.10720	
group [Prob R] * answer_day	1.78e-01	0.95	0.00691	0.3499	2.039	218	4.15e-02	0.54728	
group [Prob CC] * answer_day	-9.26e-04	0.95	-0.03655	0.0347	-0.051	218	9.59e-01	-0.00284	
group [NR] * answer_day	-3.70e-02	0.95	-0.32065	0.2466	-0.256	218	7.98e-01	-0.11356	
R2 (conditional)									0.912
R2 (marginal)									0.506

Take-home message: This model shows that LFD responders experienced, on average, an increase in their QoL by an estimated nine points relative to their intercept of 60. During reintroduction, the estimated increase was of 13 points. Both of these increases are statistically significant. Only a significant increase in QoL was observed for probiotic responders between active treatments, of an estimated seven points.

Output, Compliance

We fitted a linear mixed model (estimated using REML and nloptwrap optimizer) to predict fars with group and answer_day (formula: fars ~ group + answer_day:group). The model included random_id as random effect (formula: ~1 | random_id). The model's total explanatory power is substantial (conditional R2 = 0.63) and the part related to the fixed effects alone (marginal R2) is of 0.22. The model's intercept, corresponding to group = LFD R and answer_day = 0, is at 24.33 (95% CI [22.48, 26.19], t(287) = 25.69, *P* < .001). Within this model:

Parameter	Coefficient	CI	CI_low	CI_high	t	df_error	*P*	Std_Coefficient	Fit
(Intercept)	24.33180	0.95	22.47555	26.188037	25.691	287	0.00e+00	0.1716	
group [Re-Intro]	-3.19386	0.95	-4.60745	-1.780276	-4.428	287	9.50e-06	-0.7892	
group [Prob R]	0.39429	0.95	-2.32592	3.114505	0.284	287	7.76e-01	-0.3181	
group [LFD R] * answer_day	-0.01194	0.95	-0.06605	0.042182	-0.432	287	6.66e-01	-0.3016	
group [Re-Intro] * answer_day	-0.00319	0.95	-0.00689	0.000506	-1.692	287	9.06e-02	-0.0807	
group [Prob R] * answer_day	-0.03156	0.95	-0.07923	0.016103	-1.298	287	1.94e-01	-0.7976	
R2 (conditional)									0.633
R2 (marginal)									0.223
Sigma									2.128

This model shows that LFD responders deviated from the LFD principles during reintroduction, with an average reduction in compliance of an estimated three points relative to their intercept of 24.

Output, Bowel frequency

We fitted a linear mixed model (estimated using REML and nloptwrap optimizer) to predict frequency with group, diagnosis and bristol (formula: frequency ~ group + diagnosis + bristol). The model included random_id as random effect (formula: ~1 | random_id). The model's total explanatory power is substantial (conditional R2 = 0.61) and the part related to the fixed effects alone (marginal R2) is of 0.28. The model's intercept, corresponding to group = Baseline, diagnosis = IBS-M and bristol = 1, is at 1.37 (95% CI [0.60, 2.14], t(549) = 3.49, *P*< .001). Within this model

Parameter	Coefficient	CI	CI_low	CI_high	t	df_error	*P*	Std_Coefficient	Fit
(Intercept)	1.370	0.95	0.6011	2.1379	3.493	549	4.77e-04	-0.856	
group [LFD R]	-0.156	0.95	-0.5067	0.1945	-0.873	549	3.83e-01	-0.101	
group [Re-Intro]	-0.384	0.95	-0.6814	-0.0861	-2.527	549	1.15e-02	-0.249	
group [Prob R]	-0.531	0.95	-0.9209	-0.1406	-2.666	549	7.67e-03	-0.344	
group [Prob CC]	-0.461	0.95	-0.8456	-0.0763	-2.349	549	1.88e-02	-0.299	
group [NR]	-0.242	0.95	-0.8114	0.3264	-0.835	549	4.04e-01	-0.157	
diagnosis [IBS-D]	1.155	0.95	0.3572	1.9537	2.837	549	4.55e-03	0.749	
bristol [2]	0.625	0.95	-0.0277	1.2785	1.877	549	6.05e-02	0.406	
bristol [3]	0.726	0.95	0.1448	1.3078	2.448	549	1.44e-02	0.471	
bristol [4]	0.470	0.95	-0.0673	1.0064	1.714	549	8.65e-02	0.305	
bristol [5]	0.908	0.95	0.3699	1.4465	3.307	549	9.44e-04	0.589	
bristol [6]	1.816	0.95	1.2853	2.3474	6.704	549	0.00e+00	1.178	
bristol [7]	1.814	0.95	1.0932	2.5339	4.934	549	8.00e-07	1.176	
R2 (conditional)									0.606
R2 (marginal)									0.284
Sigma									1.032

Take-home message: This model shows that LFD responders experienced an average reduction in bowel frequency of an estimated -0.15, and during reintroduction an estimated reduction of -0.38, relative to their baseline number of 1.37 bowel movements per day. For probiotic responders, the estimated reduction was -0.53, and between active treatments it was -0.46. The latter three estimates are statistically significant.

Tables

Table 1. Instructions to patients of how often they should monitor themselves at home using the web application, IBS Constant Care

This table also includes definitions of response to treatments and consultations.

Measures	Time point
IBS-Severity Scoring System (IBS-SSS)	weekly
Bristol stool chart	daily or weekly*
Bowel frequency	daily or weekly*
Weight	weekly
IBS-Quality of Life (IBS-QoL)	once every month
Fecal calprotectin home test	twice yearly (inclusion and at one-year follow-up)
Microbiome (fecal sample)	inclusion, randomization, start and end of interventions
FODMAP adherence rating scale (FARS)	weekly during LFD and re-introduction
Medical adherence rating scale (MARS)	weekly during probiotic treatments
**Definition of response to Interventions/Treatments**	
IBS-SSS	End/start of treatment
Response	Δ ≥50 (reduction in IBS-SSS)
Symptom flare-up	Δ ≥ 50 (increase in IBS-SSS)
**Consultations**	
Physical consultations at the hospital	inclusion, randomization/ crossover (LFD/VSL#3 start), re-introduction and at one-year follow-up.
Web consultations	when needed /on demand

*Patients could home monitor their bowel frequency and stool appearance daily if they found this useful. They were instructed to measure these indices at least once weekly.

Figures

Figure 1. One-year disease course of a Low FODMAP diet responder

One-year disease course of a low FODMAP diet (LFD, four weeks) responder, including reintroduction of foods high in fermentable oligo-, di-, monosaccharides and polyols (FODMAPs) and home-monitoring using the web-application, https://ibs.constant-care.com/. IBS-SSS= irritable bowel syndrome severity scoring system. Calprotectin= fecal calprotectin. QoL= Quality of life. FARS=FODMAP adherence rating scale. Published with permission from the patient.

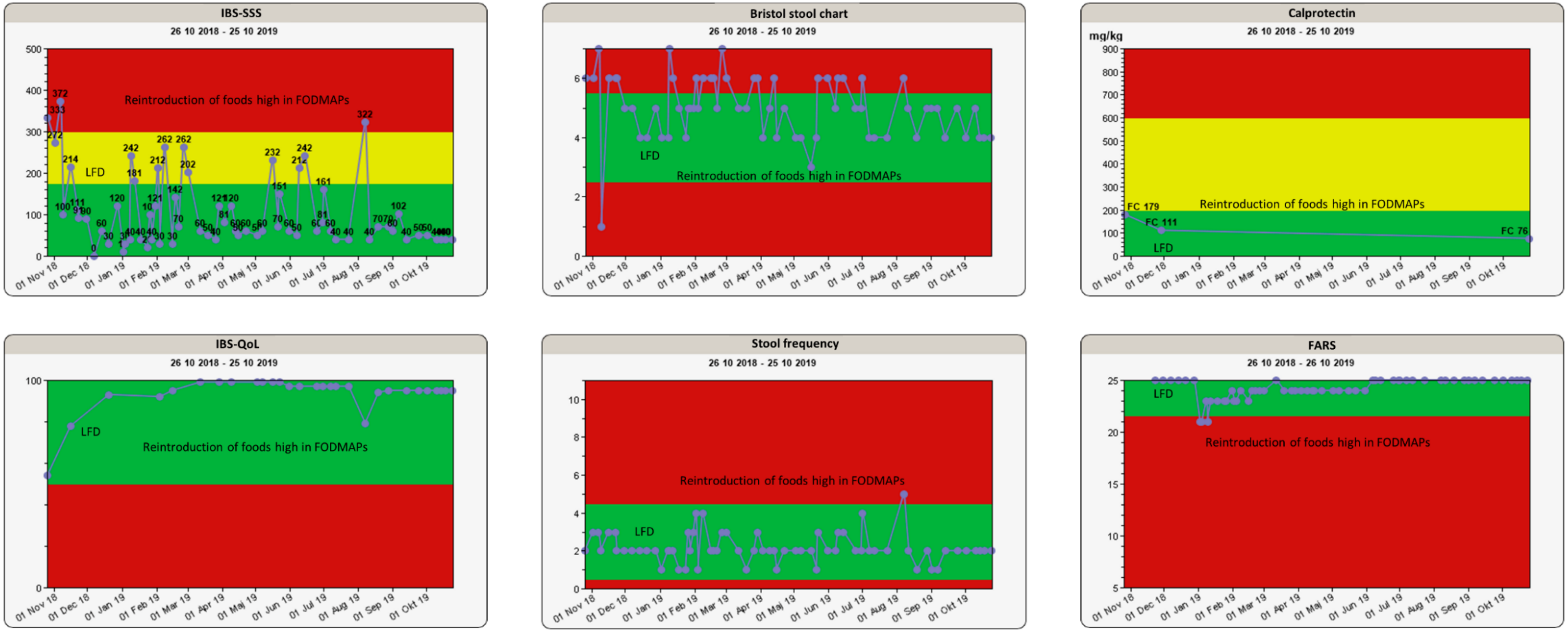

Figure 2. One-year disease course of a probiotic responder

One-year disease course of a probiotic (VSL#3®, Actial Farmaceutica s.r.l.) responder with three four-week treatments during the course of one year and home-monitoring using the web-application, https://ibs.constant-care.com/. During the study period, one participant was involved in a traffic accident and was treated at the hospital (and therefore did not receive probiotic treatments during this period). IBS-SSS= irritable bowel syndrome severity scoring system. Calprotectin= Fecal Calprotectin. QoL= Quality of life. MARS= Medical adherence rating scale. Published with permission from the patient.

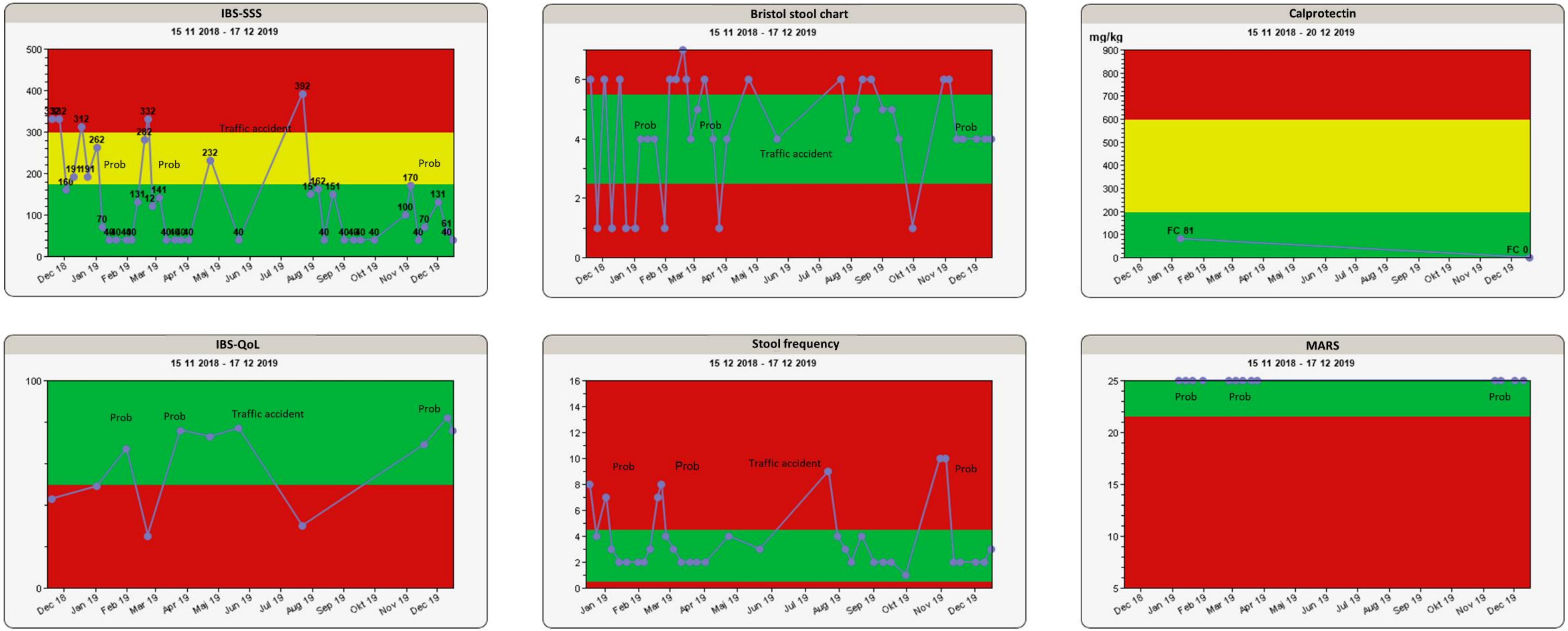

Figure 3. Change in fecal calprotectin and disease course types based on responder types

A: Change in fecal calprotectin based on responder types, and **B**: Change in disease course types (n= Prob R=7, LFD R=12, NR=4). Both figures compare baseline to one-year follow-up (1-y-FU). R= responder, NR= non-responder, LFD= low FODMAP diet, Prob = probiotic treatment. There are no significant differences between groups in Figure 3A. Missing data were unfortunately common for one-year follow-up, especially for FC measures (due to updates of the CalproSmart application some patients did not manage to complete this home test at 1-y-FU).

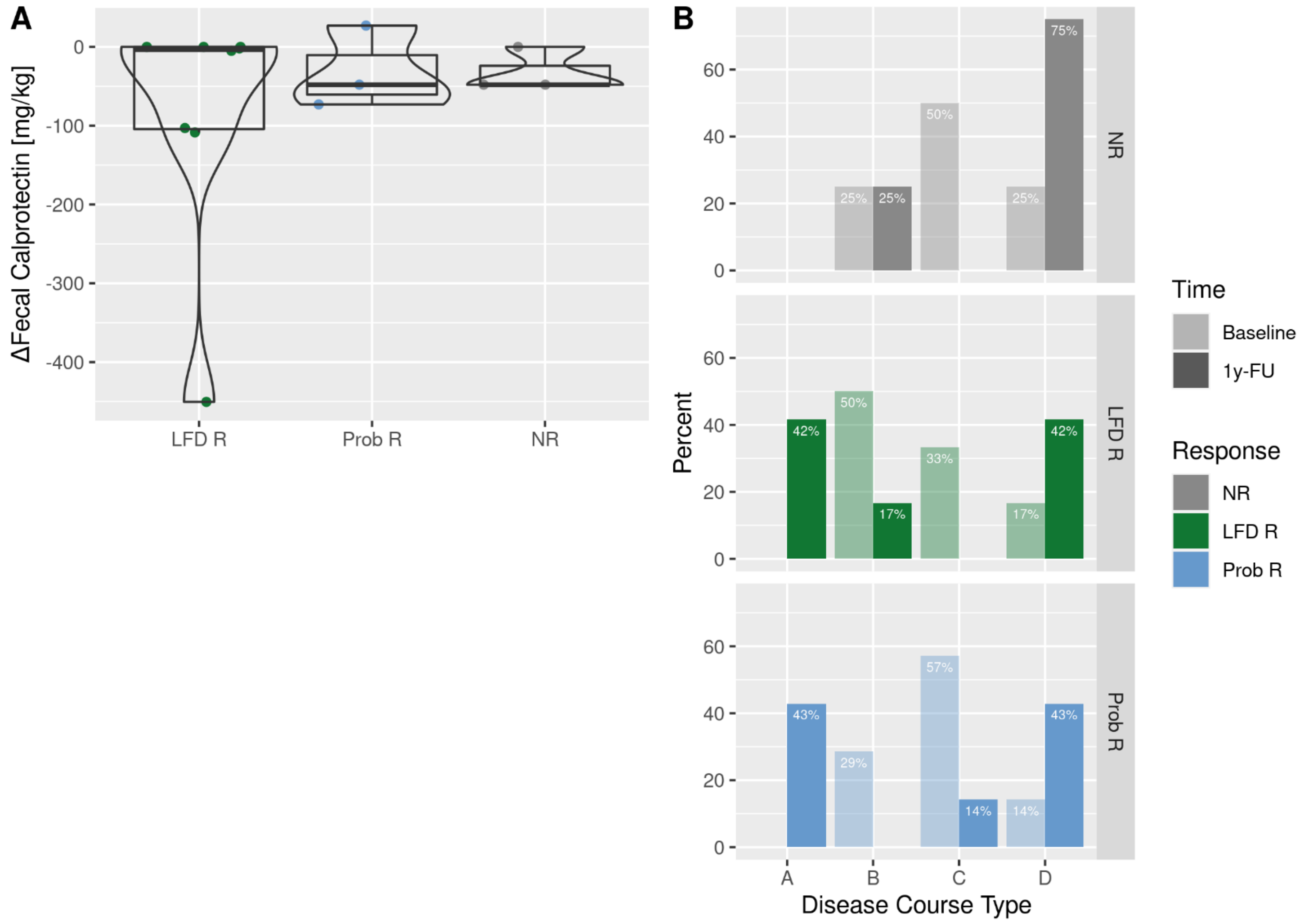

Figure 4. Patient reported evaluation at one-year follow up

Patient-reported evaluation (n=23) at one-year follow-up of the study using the web application, IBS Constant Care (https://ibs.constant-care.com/). Numbers within bars state how many of the participants who reported yes/no or the number of minutes used on average for entries in IBS CC (SD=6.1). In addition to the 10 questions in the figure, participants were also asked to comment on any challenges or recommendations for improving the web-based treatments on IBS CC. Five such common remarks (here condensed) were: 1) Easy, clear and VERY nice that you have been able to contact Dorit online if having any doubts; 2) Make IBS CC a mobile app rather than a web app. Provide a step-by-step 'for dummies' manual for when to measure what; 3) Include reminders/notifications in the app that it is time to complete forms and do a stool test; 4) It would be nice, once in a while, to take a break from the registrations and from the illness; 5) I've been happy with it all except that I was often thrown out of the web app while typing/completing forms. Extend the time allowed before logging us out of the web application.

This analysis includes the patient who did not manage to cross over to probiotic treatment after showing no response to the low FODMAP diet.

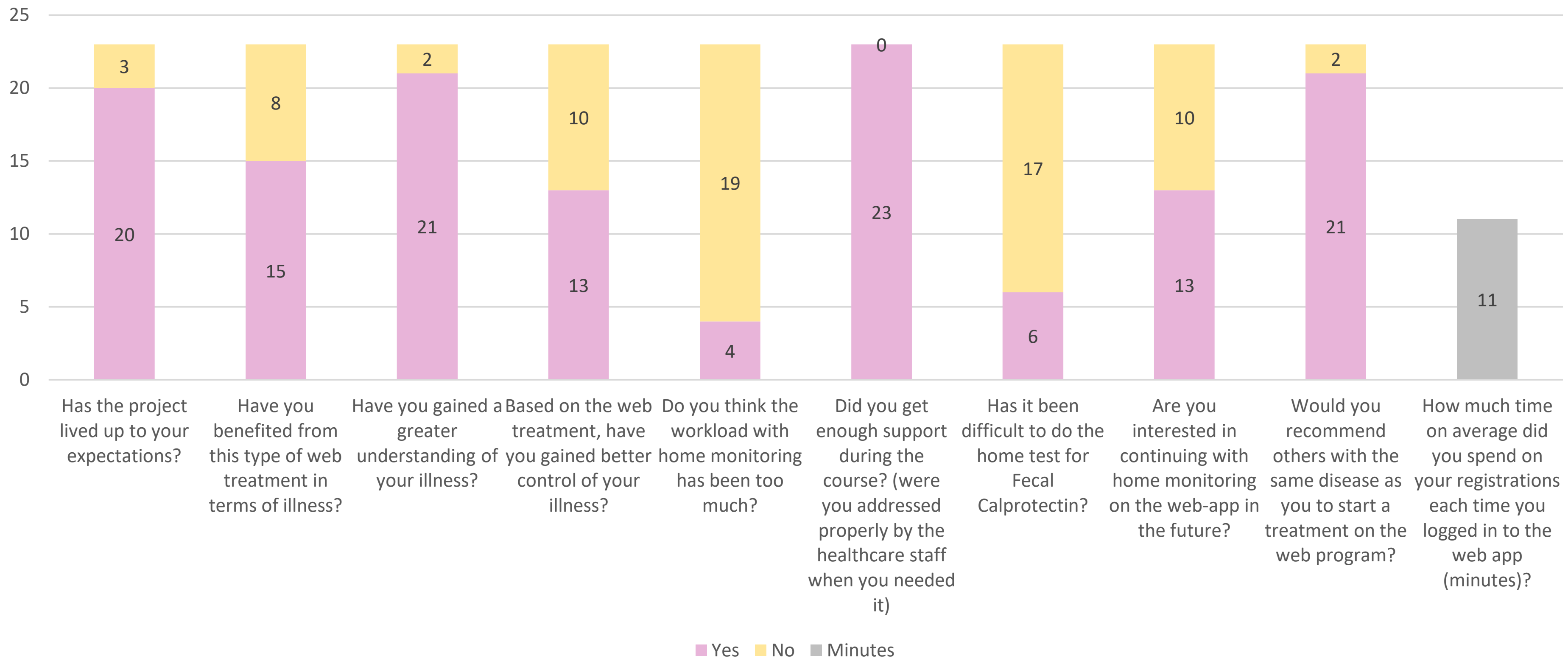

Figure 5. Alpha diversities for healthy controls and IBS patients

Alpha diversities (inverse Simpson) for healthy controls HC (n=6) and IBS patients (n=31). Each point in the figure is based on a mean of all samples from each HC, and the first two baseline samples from each IBS patient. HCs have a significantly higher median alpha diversity than IBS patients.

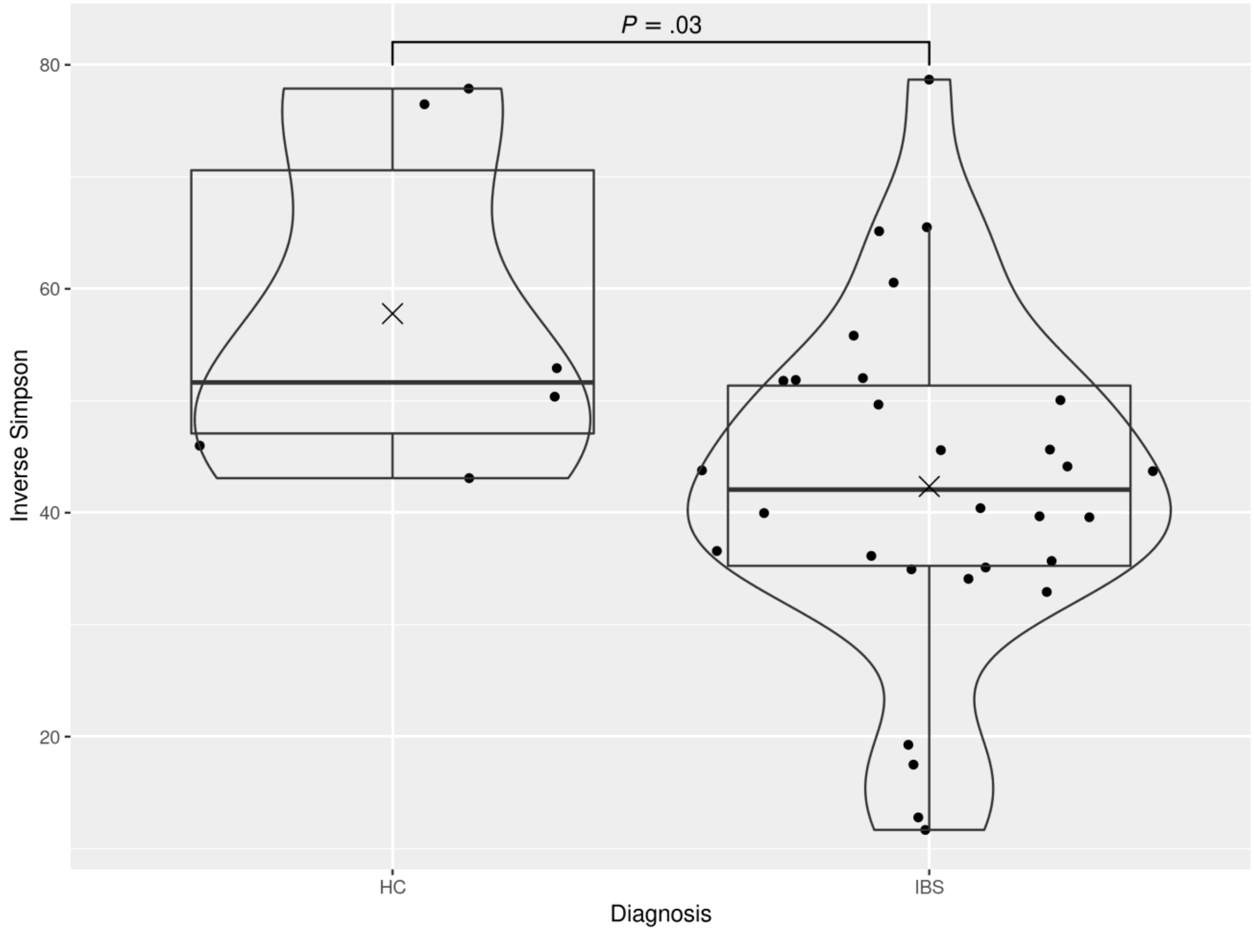

Figure 6. Inter- and intra-individual Bray Curtis dissimilarity

The inter-individual Bray-Curtis dissimilarity was significantly greater than the intra-individual dissimilarity.

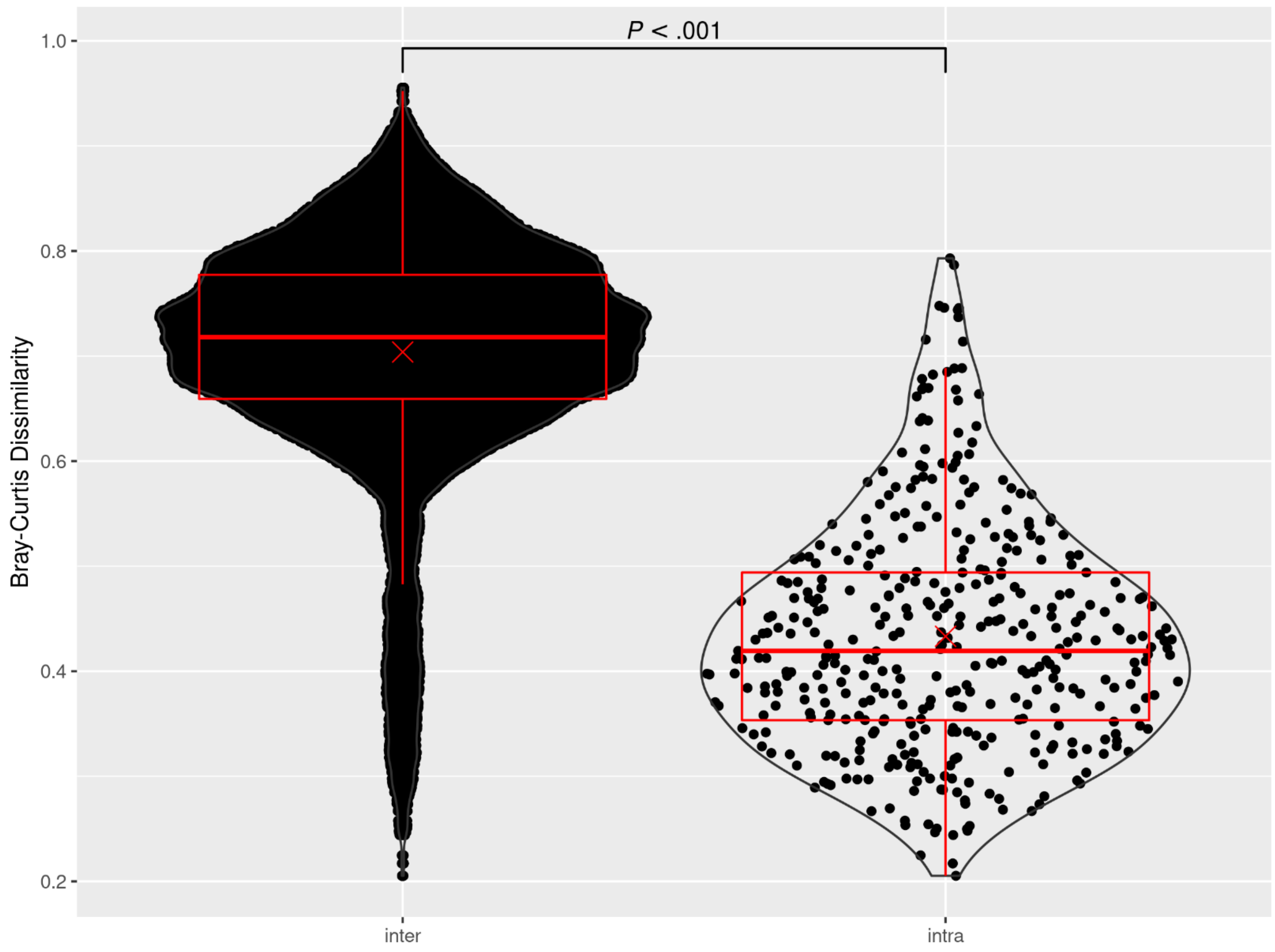

Figure 7. Intra-individual Bray-Curtis dissimilarity and UMAP plot according to diagnoses

A: Intra-individual Bray-Curtis dissimilarity according to diagnoses. **B** Uniform Manifold Approximation and Projection plot based on Bray-Curtis dissimilarity matrix of participants included in the study and colored according to IBS sub-types/diagnosis (IBS-M: n=15 and 70 samples; IBS-D: n=15 and 80 samples; healthy controls: n=6 and 23 samples). **A:** IBS-D showed a significantly higher median intra- individual Bray-Curtis dissimilarity than IBS-M (comparisons within diagnosis group: IBS-M=157, IBS-D=218, HC=33). **B:** No obvious clustering according to diagnosis can be seen in the UMAP plot.

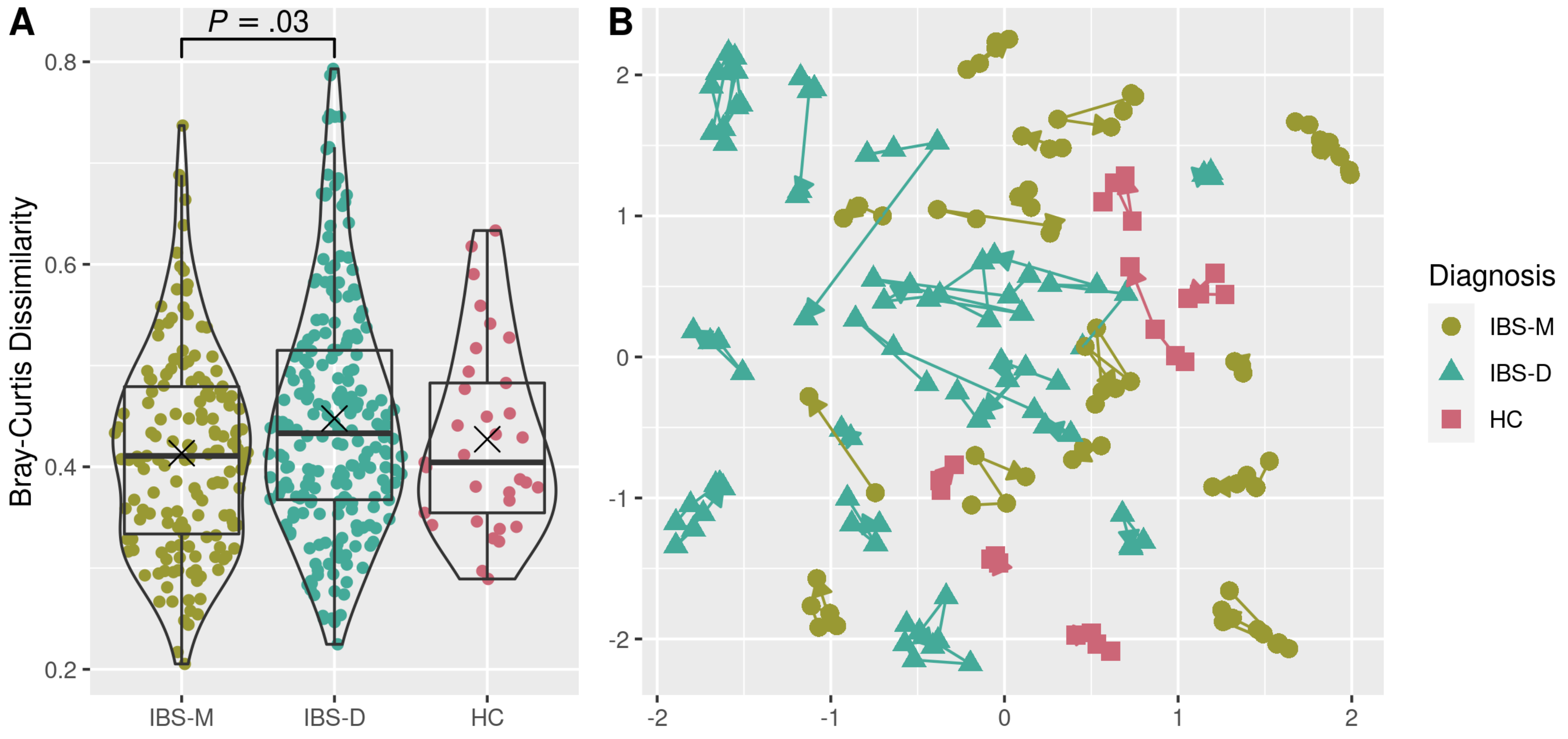

Figure 8. Intra-individual Bray-Curtis dissimilarity and UMAP plot according to responder types

A: Intra-individual Bray-Curtis dissimilarity based on responder types. **B:** UMAP plot based on Bray-Curtis dissimilarity matrix of participants included in the study and colored according to responder type (intention to treat). Low FODMAP diet (LFD) responder: n=12 and 70 samples; probiotic responder: n=12 and 80 samples; healthy controls: n=6 and 23 samples. **A:** a significantly higher median intra-individual Bray- Curtis dissimilarity was observed in probiotic responders than in Non-responders (NR), (comparisons within responder group: LFD responders=77, probiotic responders=253, Non-responders (NR)=38, HC=38). **B:** No obvious clustering of responder types can be seen in the UMAP plot.

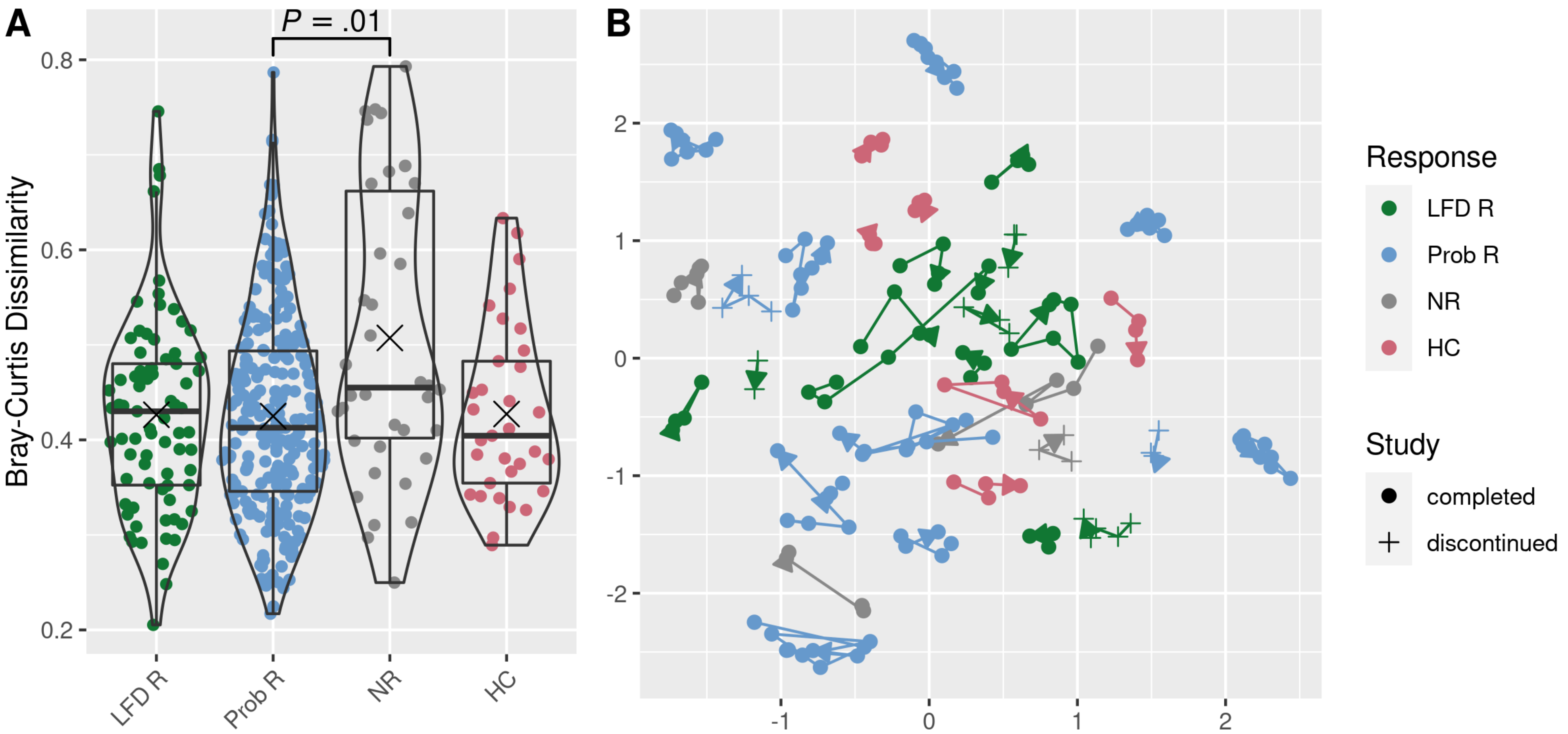

Figure 9. Alpha diversities for responder types including the corresponding output of LME

Alpha diversities (inverse Simpson) for responder types. **A:** violin plot of alpha diversities for responder types in the low FODMAP diet (LFD) arm. **B:** Output of linear mixed effect model; inverse Simpson and responder types of the LFD intervention and controlled for ID. This model shows that non-responders (NR) have a *P*-value of .07 (close to significance), which indicates that LFD non-responders (NR) showed a tendency to lose alpha diversity while dieting, whereas responders (LFD) did not show this same tendency. **C** and **D** are the corresponding figures for the probiotic intervention. No significant (<.05) results for alpha diversities were found using either method (Dunn's-test in C and linear mixed effect model in D).

A

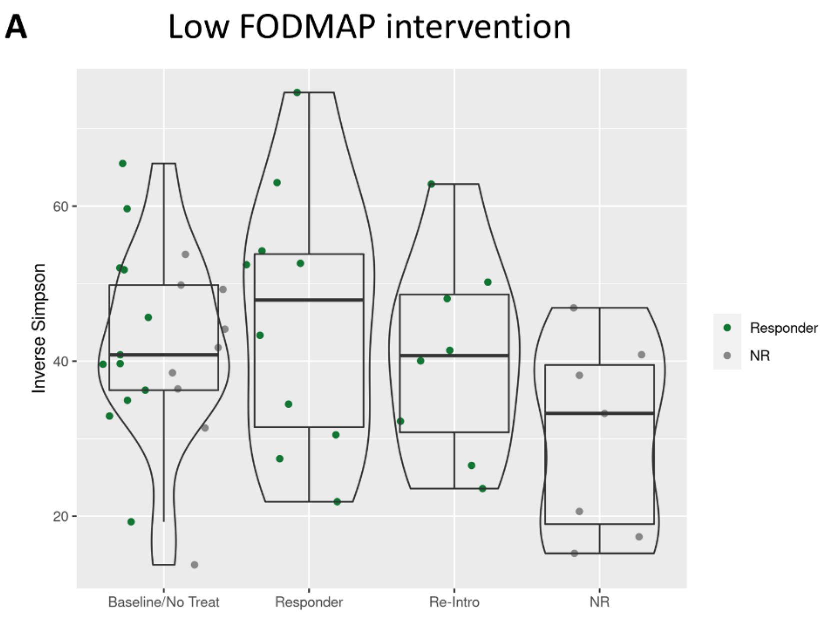

C

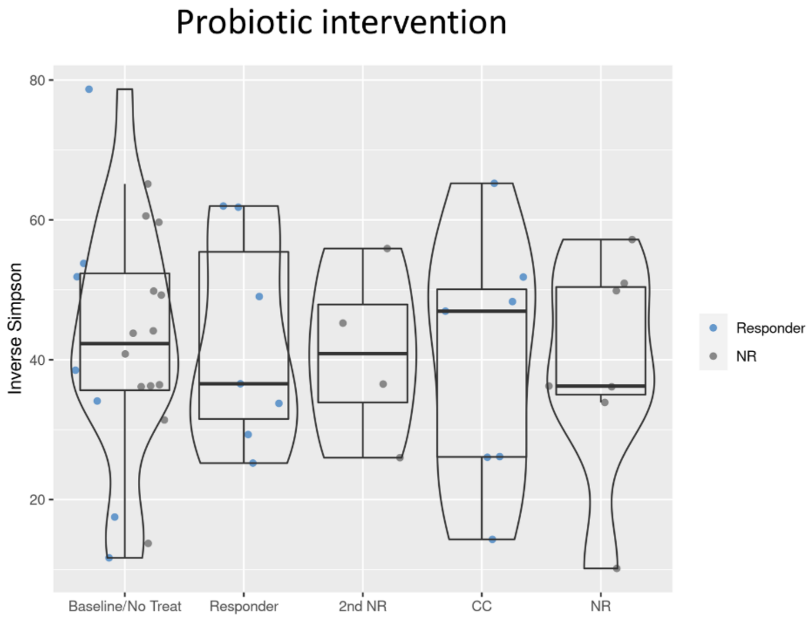

B

We fitted a linear mixed model (estimated using REML and nloptwrap optimizer) to predict inverse_simpson with group (formula: inverse_simpson ~ group). The model included random_id as random effect (formula: ~1 | random_id). The model's total explanatory power is substantial (conditional R2 = .28) and the part related to the fixed effects alone (marginal R2) is of .04. The model's intercept, corresponding to group = Baseline/No Treat, is at 41.97 (95% CI [37.27, 46.66], t(71) = 17.53, *P* < .001). Within this model:

Parameter	Coefficient	CI	CI_low	CI_high	t	df_error	p	Std_Coefficient
(Intercept)	41.97	0.95	37.27	46.661	17.525	71	0.0000	0.0473
group [Responder]	2.80	0.95	-6.23	11.831	0.608	71	0.5435	0.1887
group [Re-Intro]	-3.30	0.95	-13.30	6.702	-0.647	71	0.5179	-0.2225
group [NR]	-9.58	0.95	-20.02	0.869	-1.797	71	0.0723	-0.6457

D

We fitted a linear mixed model (estimated using REML and nloptwrap optimizer) to predict inverse_simpson with group (formula: inverse_simpson ~ group). The model included random_id as random effect (formula: ~1 | random_id). The model's total explanatory power is substantial (conditional R2 = .51) and the part related to the fixed effects alone (marginal R2) is of 5.74e-03. The model's intercept, corresponding to group = Baseline/No Treat, is at 42.43 (95% CI [35.83, 49.03], t(92) = 12.60, *P* < .001). Within this model:

Parameter	Coefficient	CI	CI_low	CI_high	t	df_error	p	Std_Coefficient
(Intercept)	42.428	0.95	35.83	49.03	12.6003	92	0.000	0.01919
group [Responder]	0.133	0.95	-8.02	8.28	0.0319	92	0.975	0.00755
group [2nd NR]	-4.643	0.95	-17.13	7.84	-0.7289	92	0.466	-0.26394
group [CC]	-2.390	0.95	-10.67	5.89	-0.5657	92	0.572	-0.13586
group [NR]	-0.790	0.95	-11.43	9.85	-0.1455	92	0.884	-0.04491
